# Acid-Catalyzed Condensation of Benzamide with Glyoxal, and Reaction Features

**DOI:** 10.3390/molecules27031094

**Published:** 2022-02-07

**Authors:** Artyom Paromov, Irina Shchurova, Alla Rogova, Irina Bagryanskaya, Dmitriy Polovyanenko

**Affiliations:** 1 Laboratory for Chemistry of Nitrogen Compounds, Institute for Problems of Chemical and Energetic Technologies, Siberian Branch of the Russian Academy of Sciences (IPCET SB RAS), 659322 Biysk, Russia; shchurova_irina@mail.ru (I.S.); alla_rogov@mail.ru (A.R.); 2 Department of Chemistry, Novosibirsk Institute of Organic Chemistry, Siberian Branch of the Russian Academy of Sciences, 630090 Novosibirsk, Russia; bagryan@nioch.nsc.ru (I.B.); dpolo@nioch.nsc.ru (D.P.)

**Keywords:** condensation, domino reactions, nitrogen heterocycles, oxygen heterocycles, 2,4,6,8,10,12-Hexaazatetracyclo[5.5.0.0^3,11^.0^5,9^]dodecane

## Abstract

Scholars from around the world have been attempting to simplify and cheapen the synthetic method for the promising high-energy compound CL-20 for decades. The lack of understanding of the formation mechanisms of hexaazaisowurtzitane derivatives―CL-20 precursors―is a barrier to solving the said problems. Here, we report the results from an in-depth study into the acid-catalyzed condensation between benzamide and glyoxal in a molar ratio of 2:1 in polar protic and aprotic solvents. Sixteen compounds were isolated and identified, of which eight were synthesized for the first time. A geminal diol, *N*,*N*’-(2,2-dihydroxyethane-1,1-diyl)dibenzamide, was synthesized. Two isomers of 1,2-bis(benzoylamino)-1,2-ethanediol were isolated and identified. *N*,*N*’-(1-oxoethane-1,2-diyl)dibenzamide and 2-oxo-2-[(phenylcarbonyl)amino]ethyl benzoate were produced that were likely formed due to the 1,2-hydride shift. *N*-polysubstituted 1,4-dioxane-2,3,5,6-tetramine was synthesized for the first time, whose structure may be of interest as a scaffold for new explosives. DMSO, THF and HCOOH were found to be able to engage in a reaction with benzamide, or condensation products thereof, and glyoxal under acid-catalyzed conditions.

## 1. Introduction

The evolution of defense technology has a direct association with developing new high-energy materials that are superior in their energy-mass and performance characteristics to the existing ones.

**Figure 1:**
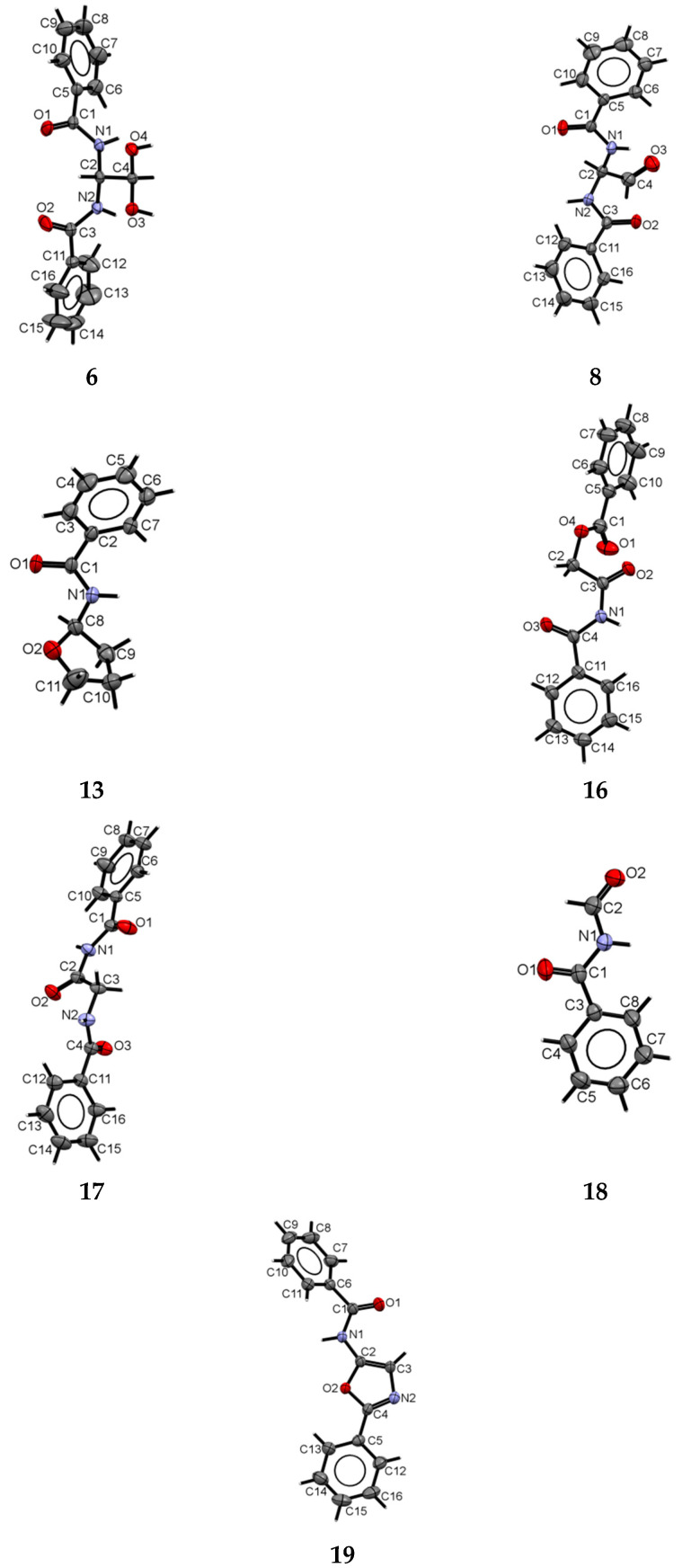
The molecular structures and atomic numbering for compounds **6**, **8**, **13** and **16**–**19** (40% thermal ellipsoids are shown).

Polyheterocyclic caged nitramines are viewed as the most promising among the high-energy density compounds. These compounds structurally contain strained moieties and have compact rigid molecules, which enhances their energy-mass characteristics. The predictions demonstrate that caged nitramines are much more attractive in seeking high-energy materials because they feature enhanced energetic characteristics and reduced sensitivity [1,2,3,4,5,6,7,8,9,10]. The density of nitramines increases with increasing molecular rigidity [11]. One notable example is hexanitro-2,4,6,8,10,12-hexaazatetracyclo[5.5.0.0^3,11^.0^5,9^]dodecane (hexanitrohexaazaisowurtzitane, CL-20) (Figure 1), which exhibits one of the highest detonation velocities (V_0_*D* = 9.36 (ε) km/s) among all of the explosives, and the highest density among the known nitranmines (ρ = 2.044 g/cm^3^) [12,13,14,15], as well as a positive enthalpy of formation (ΔH*f* = 454 kJ/mol), optimal oxygen balance (−11.0) and detonation pressure (420 kbar) [16,17,18,19,20,21]. CL-20 excels compared to the other high-energy explosive materials such as HMX, RDX, PETN, etc. CL-20 is viewed as a potential component of solid propellants [22,23,24,25,26,27] and composite explosives [28,29,30,31,32,33,34,35,36,37,38,39,40,41,42,43]. Despite the merits of CL-20, its wide application is hindered by its high manufacturing cost.

**Scheme 1:**
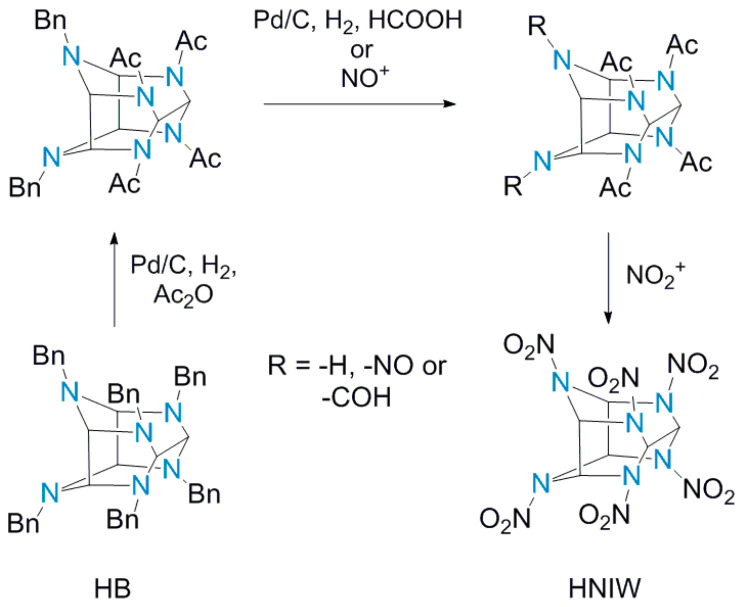
Synthesis of CL-20 via transfunctionalization of HB.

In industry, CL-20 is produced by the multistage transfunctionalization of 2,4,6,8,10,12-hexabenzyl-2,4,6,8,10,12-hexaazatetracyclo[5.5.0.0^3,11^.0^5,9^]dodecane (HB), which in turn is prepared by the condensation of benzylamine with glyoxal with about 80–90% yield [44,45] (Scheme 1).

Benzylamine, its derivatives, and similar amines bearing an aromatic moiety linked via a methylene bridge to the amino group are the only compounds that have been established to be capable of furnishing hexaazaisowurtzitane derivatives with an acceptable yield [44,45,46,47,48,49,50,51,52,53].

Despite benzylic substitution being possible [54], the direct nitration of HB to CL-20 fails.

Extensive research focused on upgrading the existing synthetic technologies for CL-20 and on finding alternative methods for obtaining the same has been pursued, dating back decades. The most promising direction is to develop a synthetic method for hexaazaisowurtzitane derivatives by direct condensation, which are nitratable to CL-20 [16,44,45,46,47,48,49,50,51,52,53,55,56,57,58,59,60,61,62,63,64].

At this point, the formation mechanism of the hexaazaisowurtzitane cage is understudied. The reasons behind it having such a high selectivity towards the structure of ammonia derivatives are unknown. It is therefore of no doubt that the most important step in designing alternative synthetic routes to CL-20 is an in-depth study into that process in order to identify new formation mechanisms of the hexaazaisowurtzitane cage, in particular the relationship between the structure/properties of the starting ammonia derivatives and their capability of being condensed with glyoxal to form hexaazaisowurtzitane derivatives.

Here, we investigated in detail the condensation between benzamide (**1**) and glyoxal in a molar ratio of 2:1 in polar protic and aprotic solvents to synthesize hexaazaisowurtzitane derivatives and establish new reaction regularities. X-ray diffraction analysis of an array of the resultant compounds was performed.

## 2. Results and Discussion

### 2.1. Study into Condensation of Benzamide with Glyoxal

We have previously explored the condensation between substituted sulfonamides (methanesulfonamide, benzenesulfonamide, 4-dimethylaminobenzenesulfonamide or isopropylsulfonamide) and glyoxal, whereby a series of new oxaazaisowurtzitane derivatives were obtained, some polyheterocyclic caged systems were discovered and new condensation regularities were established [62,63,64]. More specifically, the increased basicity of the amido group in the sulfonamide molecule was found to facilitate the incorporation of aza groups into the oxaazaisowurtzitane cage. Unlike benzenesulfonamide, 4-dimethylaminobenzenesulfonamide is condensable with glyoxal to yield an oxaazaisowurtzitane derivative bearing three aza groups. In addition, the condensation of 4-dimethylaminobenzenesulfonamide entails a byproduct that may be suggestive of the formation of a dioxatetraazaisowurtzitane derivative [64]. Gottlieb et al. proposed a probable formation pathway of a similar compound in their study [65].

In the case of the sulfonamides, limitations in the quantity of aza groups being inserted into the oxaazaisowurtzitane cage, and the low yield of the caged compounds, appear to be associated with an insufficient basicity of amido groups and steric hindrances occurring in the condensation.

Given the findings obtained earlier, we chose a compound from the class of carboxamides, namely benzamide (**1**), as the substrate for the study. The amido group in caboxamide molecules exhibits a higher basicity and steric accessibility than that in sulfonamide molecules.

One of the factors that influenced the choice of the phenyl moiety as a substituent in the molecule of carboxamide under study was the ability of the aromatic system to absorb UV light, which considerably simplifies the analysis and separation of the reaction products. In addition, the structural affinity of benzamide to benzylamine can probably have a positive effect on the formation process of hexaazaisowurtzitane derivatives. The highest yield of a hexaazaisowurtzitane derivative was achieved by the condensation between benzylamine and glyoxal [44,45].

The increased polarity of a solvent favors the progress of acid-catalyzed condensation, and therefore the condensation between benzamide and glyoxal (40%) was examined in polar protic and aprotic solvents. In all cases, the amide to glyoxal ratio was taken equal to 2:1, leading to the formation of the respective hexaazaisowurtzitane derivative. The acid catalysts used were mineral (hydrochloric (HCl) and sulphuric (H_2_SO_4_) acids) or organic (formic (HCOOH), acetic (AcOH), trifluoroacetic (TFA) and p-toluenesulfonic (PTSA)) acids. Individual compounds were isolated from the mixture by using preparative chromatography and different solubility of the resultant compounds.

First, the condensation in polar protic solvents, namely water, methyl (CH_3_OH), ethyl (CH_3_CH_2_OH) and isopropyl ((CH_3_)_2_CHOH) alcohols, was examined.

**Scheme 2:**
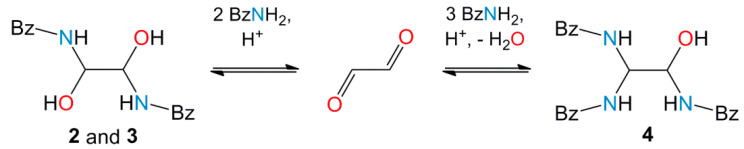
The formation of compounds **2**, **3** and **4**.

**Scheme 3:**
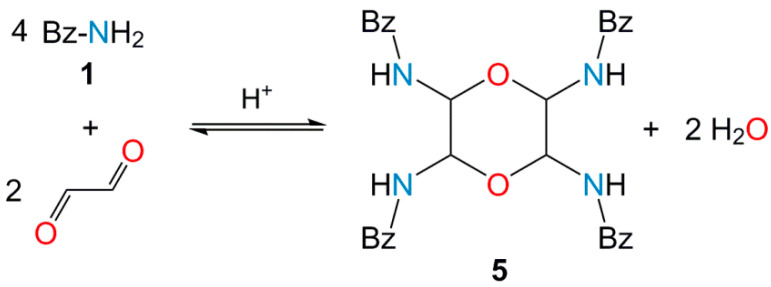
The formation of tetrabenzamide **5**.

**Scheme 4:**
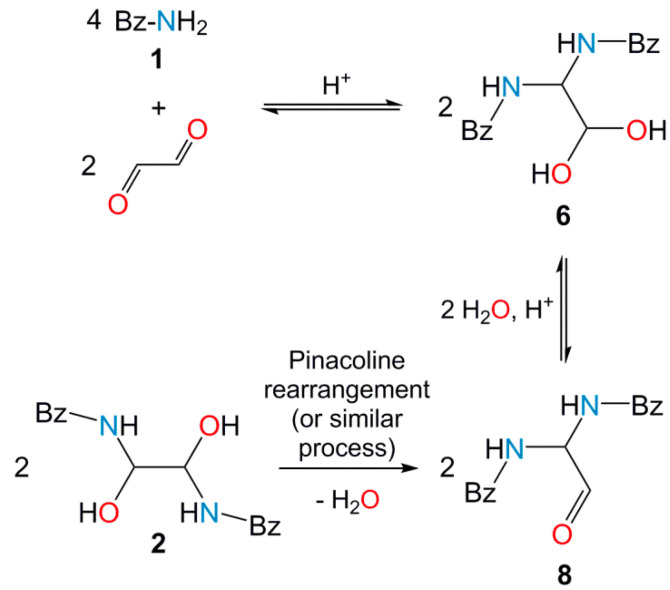
A presumed formation pathway of dibenzamides **6** and **8**.

When benzamide was condensed with glyoxal in water at room temperature over the HCl catalyst, the major reaction products were two isomeric 1,2-bis(benzoylamino)-1,2-ethanediols (**2**) and (**3**), *N*,*N*’,*N*’’-(2-hydroxyethane-1,1,2-triyl)tribenzamide (**4**), *N*,*N*’,*N*’’,*N*’’’-(1,4-dioxane-2,3,5,6-tetrayl)tetrabenzamide (**5**), *N*,*N*’-(2,2-dihydroxyethane-1,1-diyl)dibenzamide (**6**), benzoic acid (**7**) (as the benzamide hydrolysis product), and *N*,*N*’-(2-oxoethane-1,1-diyl)dibenzamide (**8**) (Scheme 2, Scheme 3 and Scheme 4).

Because the exact structures of dibenzamides **2** and **3** were not of much interest in the present study, their identification was not performed. Isomers **2** and **3** could be separated through the means of different solubility and retention times as per HPLC. The NMR spectra of these compounds were alike. However, in contrast to compound **3**, the ^1^H spectrum of **2** showed stronger spin-spin coupling between hydroxyl hydrogens and the ethane moiety (NMR spectra are included in Supplementary Materials).

The synthesis of compounds comprising the heterocyclic structure of 1,4-dioxane-2,3,5,6-tetramine has not been reported before. The high melting point (304–305 °C (dec.)) of tetrabenzamide **5** and the low solubility in protic and aprotic solvents bear mentioning. The compound is limitedly soluble in boiling dimethylsulfoxide (DMSO).

Compound **6** was probably generated by the condensation of two benzamide molecules with glyoxal, accompanied by steric hindrances. Compound **8** was likely formed by the dehydration of compound **6**. In addition, the pinacol rearrangement of diol **2** could take place (or a similar process accompanied by the 1,2-migration) to yield compound **8**, which was further hydrated to compound **6**. However, this process is unlikely. The benzoic group is less liable to the 1,2-migration. The compound bearing a 2,2-diaminoethane-1,1-diol moiety was synthesized for the first time. The structures of compounds **6** and **8** were resolved by X-ray diffraction analysis (Sub. 2.2. and Supplementary Materials). The possible pathways for the formation of dibenzamides **6** and **8** are displayed in Scheme 4.

**Scheme 5:**
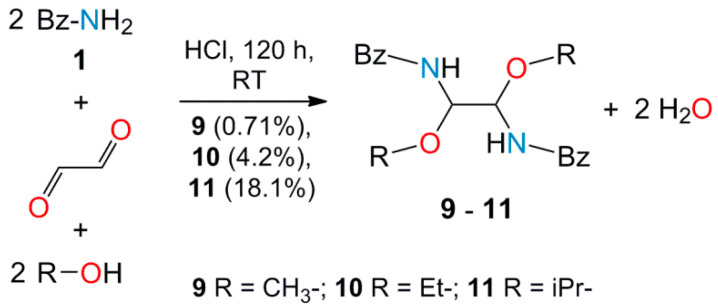
The formation of dibenzamides **9**–**11**.

The condensation in alcohols (CH_3_OH, CH_3_CH_2_OH or (CH_3_)_2_CH_2_OH), as in water, was slow and required a high acidity. The resultant products from the reaction between the alcohols and diol **2** were *N*,*N*’-(1,2-dimethoxyethane-1,2-diyl)dibenzamide (**9**), *N*,*N*’-(1,2-diethoxyethane-1,2-diyl)dibenzamide (**10**) or *N*,*N*’-(1,2-diisopropoxyethane-1,2-diyl) dibenzamide (**11**) (Scheme 5). The reaction mixture was recorded by HPLC analysis to contain a small quantity of diol **2**. Compounds **9** and **10** were previously produced by condensation of diol **2** with the corresponding alcohol over little HCl or p-toluenesulfonic acid [66]. Compound **11** was previously synthesized by the reaction between *N*,*N*’-dibenzoyl-1,2-(diaminodibromo)ethane with isopropyl alcohol [67].

Because the nucleophilic substitution rate is particularly dependent on the electronegativity of an electrophilic atom (in this case, the oxygen atom in the alcohol molecule), compound **11** was formed the quickest. All dibenzamides **9**–**11** were poorly soluble in the reaction medium and precipitated during the reaction.

Table 1 summarizes the composition of the principal reaction products of the acid-catalyzed condensation between benzamide and glyoxal over the HCl catalyst of different concentrations. The syntheses were effected for 20 h.

It is seen from the data presented in Table 1 that the condensation was the most vigorous when the HCL content in the mixture was 20%, in which case the major reaction products were **2**, **4**, **6** and **8**. A considerable portion of the initial benzamide remained in the reaction mixture.

**Scheme 6:**
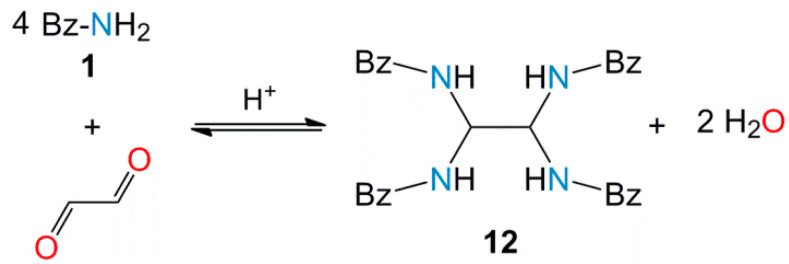
The formation of compound **12**.

The condensation between benzamide and glyoxal in polar aprotic solvents (acetone (CH_3_C(O)CH_3_), acetonitrile (CH_3_CN), tetrahydrofuran (THF) or DMSO) proceeded to furnish a complex mixture of products. In most cases, a mixture was formed that comprised more than 30 compounds. We isolated and identified most of the principal reaction products (with contents of more than 2% in the mixture, as per HPLC), namely compounds **2**–**6** and **8**, and a novel compound, *N*,*N*’,*N*’’,*N*’’’-(ethane-1,1,2,2-tetrayl)tetrabenzamide (**12**) (Scheme 6). A sample of compound **12** was obtained separately in boiling toluene over the PTSA catalyst.

**Scheme 7:**
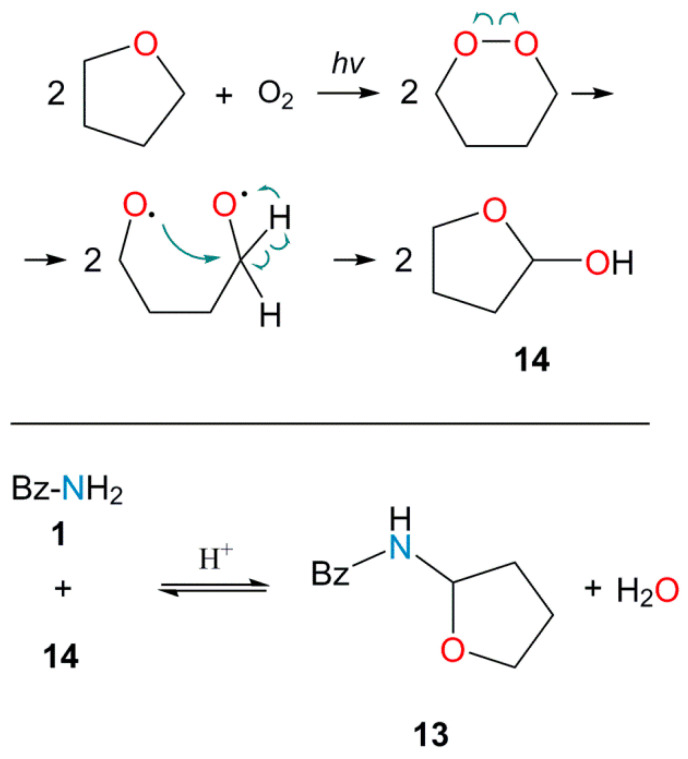
A presumed mechanism for the formation of compound **13**.

The acid-catalyzed condensation between benzamide and glyoxal in THF resulted in the formation of *N*-(tetrahydrofuran-2-yl)benzamide (**13**) (Scheme 7). This compound was most likely formed by the reaction of benzamide with the oxidized tetrahydrofuran—tetrahydrofuran-2-ol (**14**). As the process took a long time upon heating and in the light, a small amount of THF could be oxidized to 1,2-dioxane, which was transformed into compound **14** by the intramolecular transposition accompanied by the O-O bond breakage (Scheme 7). Compound **13** (5% in the mixture, as per HPLC) was formed most of all when trifluoroacetic acid was used as the acid catalyst.

**Scheme 8:**
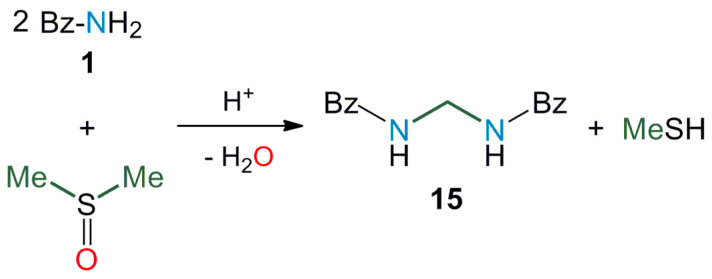
A presumed pathway for the formation of dibenzamide **15**.

A small amount of *N*,*N*’-methanediyldibenzamide (**15**) was detected to be formed by the TFA-catalyzed reaction in DMSO (Scheme 8, Table 2, Entry 7).

Because this compound was only generated in DMSO, it could be hypothesized that it was the solvent that acted as a donor of the methylene moiety in this case. The synthesis of compound **15** by the reaction of benzaldehyde, hydroxylamine, hydrochloride and DMSO has been reported. The reaction was effected in water at an elevated temperature and in the presence of alkali [68]. The authors [68] speculated that compound **15** was formed by the reaction between the methyl(methylidene)sulfonium cation and benzamide, accompanied by the formation of methanethiol (MeSH). The cation was generated in that case by the reaction of benzaldoxime with DMSO via the double bond. It can be hypothesized that a similar process accompanied by the nucleophilic attack by DMSO took place under our conditions used. After the experiment was completed, the reaction mixture had a pronounced unpleasant odor, apparently suggestive of the methanethiol formed.

Table 2 lists HPLC data for the acid-catalyzed condensation reaction between benzamide and glyoxal in polar aprotic solvents over the TFA or PTSA catalysts. These acids exhibited a high catalytic activity at an elevated synthesis temperature but did not favor intensive hydrolysis of the amide due to the lack or a small content of water (PTSA was employed as a crystallohydrate). As the condensation process was slow at room temperature, the syntheses were run at an elevated temperature (60 °C) for 4 h.

The major reaction products in CH_3_C(O)CH_3_, CH_3_CN or THF over the PTSA catalyst included compounds **4**–**6** and **8** (Table 2, Entries 1, 2 and 4). These compounds were poorly soluble in CH_3_C(O)CH_3_ and CH_3_CN and precipitated. In DMSO over the PTSA catalyst, and in CH_3_C(O)CH_3_, DMSO or THF over the TFA catalyst, compounds **2** and **3** were chiefly formed (Table 2, Entries 3, 5, 7 and 8). In CH_3_C(O)CH_3_ and THF, some of these products precipitated. In CH_3_CN over the TFA catalyst, the principal reaction products were **3**, **4**, **6** and **8** (Table 2, Entry 6). In all of the experiments, except that with DMSO, the TFA-catalyzed condensation produced a small amount of compound **12** (Table 2, Entries 1–6 and 8). In addition, minimum hydrolysis of benzamide to benzoic acid was observed in DMSO (Table 2, Entries 3 and 7). Upon completion of the syntheses, the reaction mixtures contained benzamide, and the further holding did not lead to a considerable decrement in the benzamide content, suggestive of complete glyoxal usage.

**Scheme 9:**
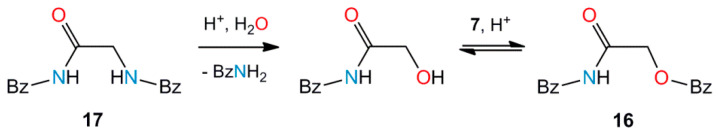
A presumed pathway for the formation of dibenzamide **16**.

**Scheme 10:**
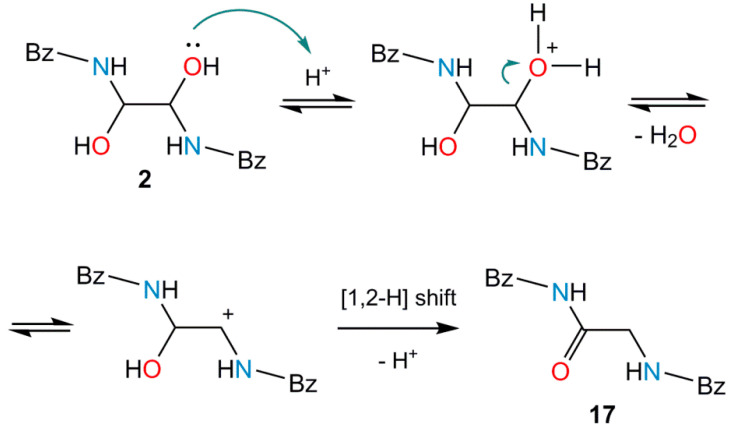
A presumed mechanism for the formation of dibenzamide **17**.

The condensation between benzamide and glyoxal was examined in strong mineral acids at reduced and room temperatures, and in organic acids at an elevated temperature. The syntheses in concentrated HCl or H_2_SO_4_ at reduced and room temperatures resulted in no formation of condensation products; instead, benzamide was observed to extensively decompose to ammonium salts. When HCl was utilized, ammonium chloride precipitated from the reaction mass. A small quantity of a new compound, 2-oxo-2-[(phenylcarbonyl)amino]ethyl benzoate (**16**), was formed in boiling HCOOH (Scheme 9). In hot TFA (60 °C), small amounts of compounds **4**, **16** and new *N*,*N*’-(1-oxoethane-1,2-diyl)dibenzamide (**17**) were generated (Scheme 10). In boiling AcOH, a complex mixture of compounds **2**, **3**, **4**, **16** and **17** originated in a small yield.

In our opinion, the synthesis of compound **17** occurred due to the acid-catalyzed transposition in the molecule of diol **2**, accompanied by the 1,2-hydride shift (Scheme 9).

Compound **16** was likely generated by the esterification reaction of the hydrolysis product of compound **17** with acid **7** (Scheme 9).

The condensation of diol **2** was explored in pure organic acids (HCOOH, TFA or AcOH) and separately in CH_3_CN or DMSO over TFA, HCl or H_2_SO_4_ catalyst (Table 3). As the reaction took place slowly at reduced and room temperatures, the syntheses were effected at an elevated temperature. Of particular interest is the ability of diol **2** to be dehydrated to the respective diimine under the acid-catalyzed condensation conditions. The formation mechanism of hexaazaisowurtzitane derivatives through the trimerization of diimines was introduced by Nielsen in 1990 [49].

**Scheme 11:**
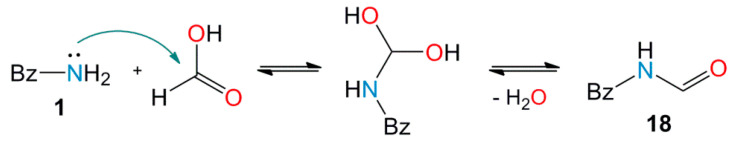
A presumed pathway for the formation of dibenzamide **18**.

A small amount of *N*-formylbenzamide (**18**) was found to be formed in boiling HCOOH, whose formation was likely due to the formylation of benzamide (Scheme 11). Since it is well-known that anhydrous HCOOH is not amenable to tautomerism (no substitution of the deuterium atom for the hydrogen atom occurs), it can be hypothesized that in this case, there was a nucleophilic attack against the carbon atom of HCOOH by the benzamide nitrogen atom, followed by dehydration of the condensation product.

**Scheme 12:**
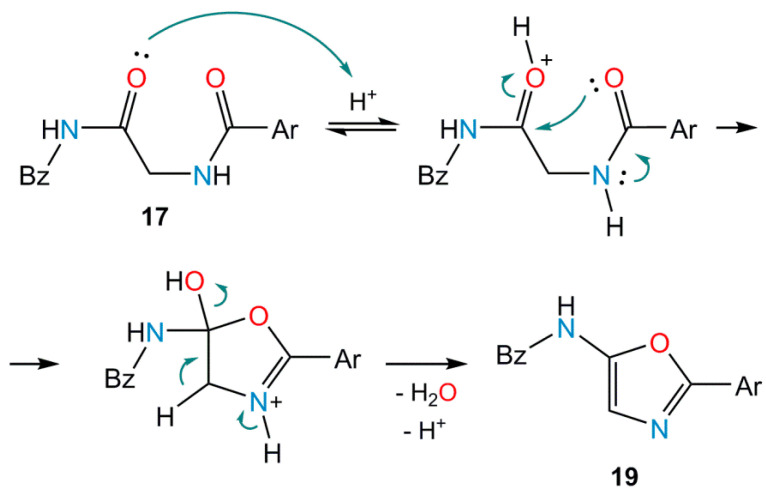
A presumed mechanism for the formation of benzamide **19**.

A novel heterocyclic compound, *N*-(2-phenyl-1,3-oxazol-5-yl)benzamide (**19**), was synthesized in boiling TFA in a 22% yield. To our minds, this compound originated from the intramolecular cyclization of compound **17**. Compound **17** was also present in the reaction mixture (Scheme 12).

Compound **17** was formed in all of the chosen media, except HCl-acidified DMSO (Table 3, Entries 1–5 and 7). Compound **16** was one of the principal reaction products in AcOH, HCOOH and TFA acids, as well as in CH_3_CN over the TFA catalyst (Table 3, Entries 1, 3 and 4). Apart from compounds **16** and **17**, a small amount of compound **18** was generated in HCOOH (Table 3, Entry 1). The principal reaction product in TFA was benzamide **19** (Table 3, Entry 2). The major reaction product in DMSO acidified with strong mineral acids was byproduct **15** (up to a 50% content in the mixture) (Table 3, Entries 6 and 7).

Table 3 presents data on the transformation of diol **2** in organic acids and acidified polar aprotic solvents at an elevated temperature.

### 2.2. X-ray Diffraction Analysis

XRD data were collected on a Bruker Kappa Apex II CCD diffractometer using φ,ω-scans of narrow (0.5°) frames with MoKα radiation (λ = 0.71073 Å) and a graphite monochromator at room temperature. The structures were resolved by direct methods in SHELXL97 [69] and refined by a full matrix least-squares anisotropic-isotropic (for H atoms) procedure using SHELXL-2014/7 software package [70]. Absorption corrections were applied using the empirical multiscan method with the SADABS program [71]. The positions of the hydrogen atoms were calculated with the riding model. The hydrogen atom positions for NH groups were located from the difference Fourier map.

CCDCs 2108741 (for **6**), 2108742 (for **8**), 2108743 (for **13**), 2108744 (for **16**), 2108745 (for **17**), 2108746 (for **18**) and 2108747 (for **19**) contain supplementary crystallographic data for this paper. These data can be obtained free of charge from the Cambridge Crystallographic Data Center at http://www.ccdc.cam.ac.uk/data_request/cif. (accessed on 28 January 2022).

The molecular structures of compounds **6**, **8**, **13** and **16**–**19** are illustrated in Figure 1. The obtained crystal structures were analyzed for the geometrical parameters and short contacts between non-bonded atoms using the PLATON [72,73] and MERCURY programs [74]. The geometric parameters for all the compounds agreed within 3σ of the corresponding mean statistical values [75].

The structure of compound **8** is made up of two crystalographically independent molecules. Crystal packings of molecules **6**, **8**, **13** and **16**–**19** are very close. The 1D infinite chains of the molecules were formed in crystals **8**, **13**, **16** and **19** through intermolecular H-bonding: N-H…O or N-H…N types. The 1D infinite ribbons were formed in crystal packings of **6** and **17** by their two infinite chains connected in pairs through intermolecular H-bonding: N-H…O or O-H…O types. Dimers were formed in compound **18** by intermolecular H-bonding.

The detailed parameters of intermolecular H-bonding are given in Table 4.

**Figure 2:**
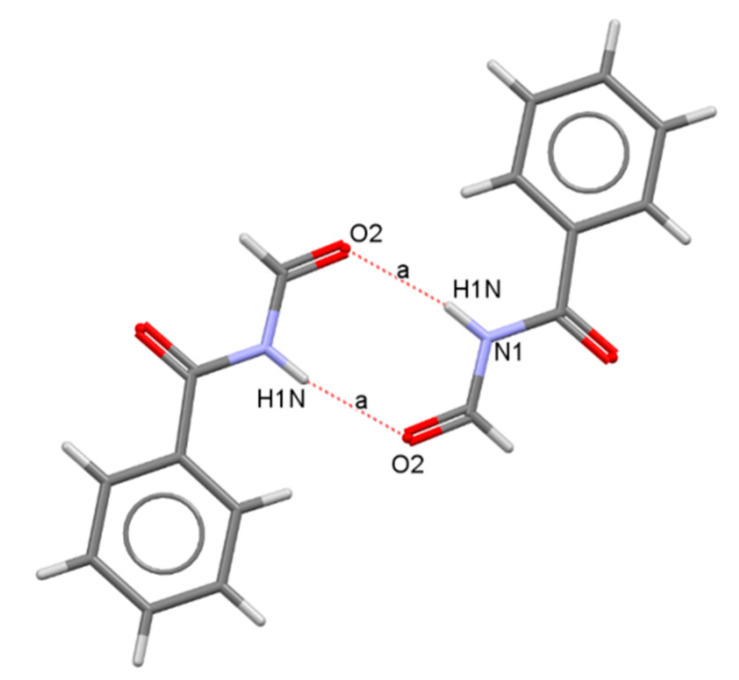
An example of the dimer in crystal packings of compound **18**.

**Figure 3:**
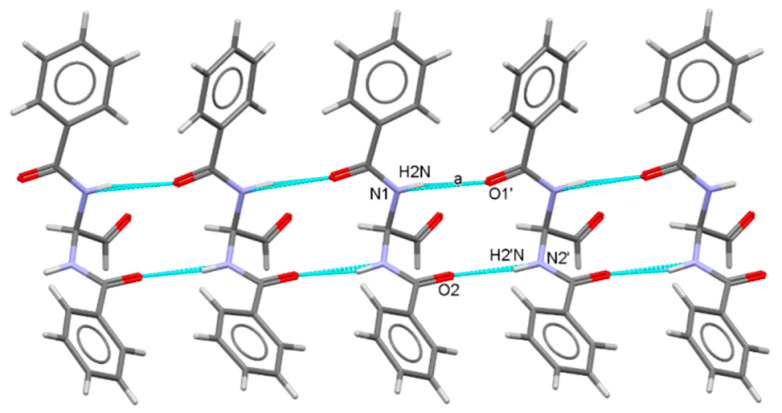
Examples of the chain of compound **8**.

**Figure 4:**
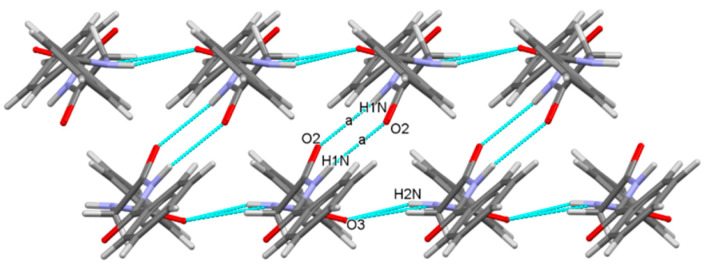
Examples of the chain of compound **17**.

For example, the dimer in crystal packings of **18**, the chain of **8** and the ribbon of **17** are presented in Figure 2, Figure 3 and Figure 4.

Figure 2, Figure 3 and Figure 4 display examples of a dimer in crystal packings of **18**, a chain of **8**, and a ribbon of **17**.

The crystallographic data and details of the refinements are given in the Supplementary Materials.

## 3. Materials and Methods

The reagents were purchased from commercial sources and were used as received, unless mentioned otherwise. Commercially available compounds were used without further purification, unless stated otherwise. Melting points were determined on a Stuart SMP30 melting point apparatus (Bibby Scientific Ltd., Staffodshire, UK). Infrared (IR) spectra were recorded on a Simex FT-801 Fourier transform infrared spectrometer in KBr pellets or in a liquid film. ^1^H and ^13^C NMR spectra were recorded on a Bruker AV-400 instrument (Bruker Corporation, Billerica, MA, USA) at 400 and 100 MHz. Chemical shifts are expressed in ppm (δ). Elemental analysis was performed on a ThermoFisher FlashEA 1112 elemental analyzer (ThermoFisher, Waltham, MA, USA). For preparative chromatography, silica gel Kieselgel 60 (0.063–0.2 mm, Macherey-Nagel GmbH & Co. KG, Dueren, Germany) was used. HPLC analysis was performed on an Agilent 1200 chromatograph with a diode array detector. The separation was carried out on a Zorbax SB-C18 (150 × 2.1 mm, a 5 µm mesh) column with a Zorbax SB-C18 pre-column (12.5 × 2.1 mm, a 5 µm mesh). Mixed solvents A (37.5 мM ammonium acetate solution, pH = 4.4 (acetic acid)) and B (acetonitrile) were used as the mobile phase. The mobile phase composition was varied in the gradient mode: the concentration of solvent B was linearly raised from 15 to 25% for 10 min, and then the concentration of solvent B was raised from 25 to 70% for 15 min and maintained at this level for another 10 min. The flowrate of the eluent was 0.25 mL/min, the column temperature was 40 °C, detection was run at a 235 nm wavelength, and the sample volume was 2 µL. The column conditioning time between successive injections was 15 min, and the total time of analysis was 50 min.

## 4. Experimental

### 4.1. Synthesis of 1,2-Bis(benzoylamino)-1,2-ethanediol (***2***) (Isomer 1)

Into a mixture of **1** (77.5 g, 0.640 mol), glyoxal (46.4 g, 40%, 0.320 mol) and DMSO (542.5 mL) was poured HCl (387.5 mL, 36%) for 3–5 min with stirring and cooling. During the portionwise addition, the reaction mixture temperature must not exceed 30 °C. After the portionwise addition was completed, the mixture was stirred at room temperature for 72 h, and afterwards it was filtered. The filter cake was washed thrice with water and dried at room temperature to a constant weight to furnish diol **2** as a white crystalline powder.

Yield: 46.5 g (96.5% assay (HPLC)), 0.149 mol (46.7% calculated as compound **1**). Mp = 178–179 °C (dec.). IR (KBr): ν = 3339, 3267, 3085, 3059, 3033, 2961, 2827, 2697, 1649, 1604, 1581, 1556, 1492, 1421, 1349, 1291, 1241, 1190, 1158, 1074, 1034, 1021, 923, 883, 800, 702, 685 cm^−1^. ^1^H NMR (DMSO-d6): δ = 5.50–5.55 (m, 2H), 5.95 (d, J = 4.5 Hz, 2H), 7.48 (t, J = 7.4 Hz, 3H), 7.54 (t, J = 7.2 Hz, 3H), 7.92 (d, J = 7.2 Hz, 4H), 8.69 (d, J = 7.8 Hz, 2H) ppm. ^13^C{1H} NMR (DMSO-d6): δ = 75.3, 127.9, 128.7, 131.8, 134.9, 166.6 ppm. Elemental analysis, calcd (%) for C_16_H_16_N_2_O_4_ (300.31): C, 63.99; H, 5.37; N, 9.33; found: C, 63.82; H, 5.44; N, 9.21.

### 4.2. Synthesis of N,N’,N’’-(2-Hydroxyethane-1,1,2-triyl)tribenzamide (***4***)

A mixture of **1** (3 g, 24.76 mmol), glyoxal (1.8 g, 40%, 12.40 mmol) and formic acid (25 mL) was stirred at reflux for 16 h, whereupon HCOOH was withdrawn from the reaction mass in a rotary evaporator at reduced pressure. The residue was muddled in acetone (30 mL) for 1 h and then collected by filtration. The resulting precipitate was recrystallized thrice from 3:1 *v/v* mixed acetone and water to furnish compound **4** as a white crystalline powder.

Yield: 0.17 g (97.1% assay (HPLC)), 0.41 mmol (5% calculated as compound **1**). Mp = 190–192 °C (dec.). IR (KBr): ν = 3298, 3112, 3087, 3063, 3030, 2919, 1653, 1603, 1579, 1558, 1527, 1487, 1351, 1278, 1156, 1075, 1060, 1040, 1013, 948, 926, 885, 802, 694, 673 cm^−1^. ^1^H NMR (DMSO-d6): δ = 5.82–5.86 (m, 1H), 6.10 (q, J_1_ = 13.0 Hz, J_2_ = 7.4 Hz, 1H), 6.37 (d, J = 4.2 Hz, 1H), 7.46–7.55 (m, 9H), 7.86 (d, J = 7.0 Hz, 6H), 8,57 (t, J = 7.9 Hz, 2H), 8.89 (d, J = 8.7 Hz, 1H) ppm. ^13^C{1H} NMR (DMSO-d6): δ = 60.3, 74.1, 127.8, 127.9, 128.73, 128.79, 131.9, 132.0, 134.7, 134.9, 166.3, 166.7, 166.9 ppm. Elemental analysis, calcd (%) for C_23_H_21_N_3_O_4_ (403.43): C, 68.47; H, 5.25; N, 10.42; found: C, 68.59; H, 5.28; N, 10.31.

### 4.3. Synthesis of N,N’,N’’,N’’’-(1,4-Dioxane-2,3,5,6-tetrayl)tetrabenzamide (***5***)

A mixture of **1** (10 g, 82.55 mmol), glyoxal (6 g, 40%, 41.35 mmol), THF (100 mL) and PTSA monohydrate (5 g) was stirred vigorously at 60 °C for 4 h. Upon completion, the mixture was filtered and the filter cake washed with THF. The residue was transferred into a beaker and successively washed thrice with hot aqueous acetone (50%) and twice with hot DMF and once with acetone for 20 min with stirring. The solvents were decanted each time. After being washed, the residue was air-dried to a constant weight to give compound **5** as a white crystalline powder.

Yield: 0.87 g (98.7% assay (HPLC)), 1.52 mmol (7.4% calculated as compound **1**). Mp = 304–305 °C (dec.). IR (KBr): ν = 3302, 3059, 2927, 1657, 1603, 1581, 1529, 1491, 1325, 1283, 1248, 1183, 1159, 1126, 1071, 1028, 1001, 927, 881, 854, 802, 692, 649 cm^−1^. ^1^H NMR (DMSO-d6): δ = 5.71 (d, J = 6.1 Hz, 4H), 7.45 (t, J = 7.2 Hz, 8H), 7.53 (t, J = 7.0 Hz, 4H), 7.84 (d, J = 7.5 Hz, 8H), 9.30 (s, 4H) ppm. ^13^C{1H} NMR (DMSO-d6): δ = 77.5, 128.0, 128.7, 132.2, 134.3, 167.1 ppm. Elemental analysis, calcd (%) for C_32_H_28_N_4_O_6_ (564.59): C, 68.07; H, 5.00; N, 9.92; found: C, 69.71; H, 5.08; N, 9.89.

### 4.4. Synthesis of Mixed N,N’-(2,2-Dihydroxyethane-1,1-diyl)dibenzamide (***6***) and N,N’-(2-Oxoethane-1,1-diyl)dibenzamide (***8***)

A mixture of **1** (8 g, 66.04 mmol), glyoxal (4.8 g, 40%, 33.08 mmol), water (16 mL) and HCl (16 mL, 36%) was stirred at room temperature for 70 h and then filtered. The stock solution when stirred was diluted with water (500 mL), held at 3–5 °C for 16 h and then filtered. The resultant precipitate (containing 70% mixed compounds **6** and **8**, as per HPLC) were recrystallized thrice from mixed acetone/water in a ratio of 4:1 *v/v* to furnish mixed compounds **6** and **8** as a white crystalline powder.

Yield: 0.39 g (the content of **6** and **8** in the precipitate was 93% (HPLC)); the contents of compounds **6** and **8** in the mixture were 36% and 64%, respectively (as per ^1^H NMR).

The crystals of compounds **6** and **8** for X-ray diffraction were derived by multiple fractional crystallization in acetonitrile (Sub. 2.2. and Supplementary Materials).

### 4.5. A General Synthetic Procedure for N,N’-(1,2-Dimethoxyethane-1,2-diyl)dibenzamide (***9***), N,N’-(1,2-Diethoxyethane-1,2-diyl)dibenzamide (***10***) and N,N’-(1,2-Diisopropoxyethane-1,2-diyl)dibenzamide (***11***)

A mixture of **1** (3 g, 24.76 mmol), glyoxal (1.8 g, 40%, 12.40 mmol), alcohol (CH_3_OH, CH_3_CH_2_OH or (CH_3_)_2_CH_2_OH) (30 mL) and HCl (5 mL, 36%) was stirred at room temperature for 120 h. Upon completion, the mixture was filtered, and the filter cake was washed thrice with water and dried at room temperature to a constant weight to yield compounds **9**, **10** or **11**, respectively, as white crystalline powders.

Yield of **9**: 0.03 g (95% assay (HPLC)), 0.087 mmol (0.7% calculated as compound **1**). Mp = 233–234 °C (dec.). IR (KBr): ν = 3335, 3061, 2985, 2950, 2934, 2835, 1645, 1603, 1520, 1489, 1462, 1450, 1353, 1327, 1302, 1249, 1189, 1150, 1099, 1074, 1046, 1002, 941, 881, 802, 718, 689, 606 cm^−1^. ^1^H NMR (DMSO-d6): δ = 3.27 (s, 6H), 5.46 (d, J = 6.8 Hz, 2H), 7.51 (t, J = 7.3 Hz, 4H), 7.58 (t, J = 7.0 Hz, 2H), 7.96 (d, J = 7.4 Hz, 4H), 8.94 (d, J = 6.7 Hz, 2H) ppm. ^13^C{1H} NMR (DMSO-d6): δ = 55.5, 81.2, 128.0, 128.8, 132.0, 134.4, 167.5 ppm. Elemental analysis, calcd (%) for C_18_H_20_N_2_O_4_ (328.36): C, 65.84; H, 6.14; N, 8.53; found: C, 65.69; H, 6.13; N, 8.50.

Yield of **10**: 0.2 g (93% assay (HPLC)), 0.52 mmol (4.2% calculated as compound **1**). Mp = 239–240 °C (dec.) (DMF). IR (KBr): ν = 3332, 3062, 3031, 2990, 2971, 2929, 2899, 2890, 1644, 1603, 1580, 1520, 1489, 1345, 1317, 1244, 1180, 1150, 1092, 1041, 1001, 922, 858, 802, 719, 691, 659, 619, 606 cm^−1^. ^1^H NMR (DMSO-d6): δ = 1.05 (t, J = 6.9 Hz, 6H), 3.49–3.64 (m, 4H), 5.52 (d, J = 6.9 Hz, 2H), 7.50 (t, J = 7.4 Hz, 4H), 7.56 (t, J = 7.1 Hz, 2H), 7.94 (d, J = 7.7 Hz, 4H), 8.94 (d, J = 7.0 Hz, 2H) ppm. ^13^C{1H} NMR (DMSO-d6): δ = 15.6, 63.3, 80.1, 127.9, 128.8, 131.9, 134.6, 167.4 ppm. Elemental analysis, calcd (%) for C_20_H_24_N_2_O_4_ (356.41): C, 67.40; H, 6.79; N, 7.86; found: C, 67.34; H, 6.82; N, 7.83.

Yield of **11**: 1.0 g (86% assay (HPLC)), 2.24 mmol (18.1% calculated as compound **1**). Mp = 246–247 °C (dec.) (DMF). IR (KBr): ν = 3331, 3062, 2972, 2929, 2900, 2873, 1644, 1602, 1580, 1524, 1491, 1456, 1378, 1365, 1343, 1311, 1245, 1179, 1152, 1135, 1120, 1089, 1042, 923, 881, 818, 799, 720, 693, 662, 622 cm^−1^. ^1^H NMR (DMSO-d6): δ = 1.05 (dd, J_1_ = 18.9 Hz, J_2_ = 6.0 Hz, 12H), 3.79–3.88 (m, 2H), 5.53 (d, J = 7.0 Hz, 2H), 7.49 (t, J = 7.4 Hz, 4H), 7.55 (t, J = 7.0 Hz, 2H), 7.94 (d, J = 7.7 Hz, 4H), 8.95 (d, J = 7.3 Hz, 2H) ppm. ^13^C{1H} NMR (DMSO-d6): δ = 22.4, 23.6, 69.8, 79.2, 127.9, 128.8, 131.9, 134.7, 166.9 ppm. Elemental analysis, calcd (%) for C_22_H_28_N_2_O_4_ (384.47): C, 68.73; H, 7.34; N, 7.29; found: C, 68.65; H, 7.37; N, 7.26.

### 4.6. Synthesis of N,N’,N’’,N’’’-(Ethane-1,1,2,2-tetrayl)tetrabenzamide (***12***)

A mixture of **1** (2 g, 16.51 mmol), glyoxal (1.2 g, 40%, 8.27 mmol), toluene (25 mL) and PTSA monohydrate (0.15 g) was refluxed with vigorous stirring in a round-bottom flask fitted with a Dean–Stark trap for 7.5 h. Upon completion, the whole mixture was filtered, and the filter cake was transferred into a beaker and muddled in ethanol (30 mL, 96%) for 1 h. The mixture was then filtered and the filter cake was washed with acetone. The resultant residue was recrystallized twice from a little DMSO to deliver compound **12** as a white crystalline powder.

Yield: 0.33 g (97.1% assay (HPLC)), 0.63 mmol (15.3% calculated as compound **1**). Mp = 325–327 °C. IR (KBr): ν = 3286, 3103, 3061, 3028, 2925, 2855, 1655, 1603, 1580, 1546, 1516, 1488, 1446, 1358, 1333, 1294, 1277, 1154, 1075, 1057, 1038, 1025, 1001, 925, 889, 880, 800, 704, 693, 673, 615 cm^−1^. ^1^H NMR (DMSO-d6): δ = 6.34–6.40 (m, 2H), 7.48 (t, J = 7.3 Hz, 8H), 7.55 (t, J = 7.0 Hz, 4H), 7.77 (d, J = 7.5 Hz, 8H), 8.63 (d, J = 6.6 Hz, 4H) ppm. ^13^C{1H} NMR (DMSO-d6): δ = 59.8, 127.5, 129.0, 132.1, 134.4, 166.6 ppm. Elemental analysis, calcd (%) for C_30_H_26_N_4_O_4_ (506.55): C, 71.13; H, 5.17; N, 11.06; found: C, 70.85; H, 5.15; N, 11.00.

### 4.7. Synthesis of N,N’-Methanediyldibenzamide (***15***)

A mixture of diol **2** (1.5 g, 4.99 mmol), DMSO (50 mL) and HCl (1 mL, 36%) was stirred at 115–118 °C for 7 h. Upon completion, the whole mixture was diluted with water (450 mL) with stirring, held for 16 h at 3–5 °C and filtered. The resultant precipitate was dissolved in chloroform and collected by filtration through a paper filter. The filter cake was washed with chloroform. The stock solution and wash chloroform were combined and evaporated to dryness in a rotary evaporator at reduced pressure. The residue was recrystallized from chloroform to deliver compound **15** as a white crystalline powder.

Yield: 0.42 g (97.6% assay (HPLC)), 1.61 mmol (32.3% calculated as compound **1**). Mp = 221–223 °C. IR (KBr): ν = 3306, 3059, 2967, 1634, 1578, 1527, 1488, 1389, 1326, 1304, 1288, 1151, 1111, 1032, 728, 694, 672 cm^−1^. ^1^H NMR (DMSO-d6): δ = 4.88 (t, J = 5.6 Hz, 2H), 7.46 (t, J = 7.4 Hz, 4H), 7.53 (t, J = 7.2 Hz, 2H), 7.92 (d, J = 7.4 Hz, 4H), 9.08 (t, J = 5.3 Hz, 2H) ppm. ^13^C{1H} NMR (DMSO-d6): δ = 45.6, 127.9, 128.7, 131.9, 134.4, 166.9 ppm. Elemental analysis, calcd (%) for C_23_H_21_N_3_O_4_ (403.43): C, 68.47; H, 5.25; N, 10.42; found: C, 68.59; H, 5.28; N, 10.31.

### 4.8. Synthesis of 2-Oxo-2-[(phenylcarbonyl)amino]ethyl benzoate (***16***) and N,N’-(1-Oxoethane-1,2-diyl)dibenzamide (***17***)

A mixture of diol **2** (9 g, 29.97 mmol) and HCOOH (90 mL) was refluxed for 1 h. Upon completion, the whole was diluted with water (810 mL) upon stirring, held for 16 h at 3–5 °C and filtered. The resulting precipitate was dissolved in chloroform and collected by filtration through a paper filter. The filter cake was washed with chloroform. The stock solution and wash chloroform were combined and evaporated to dryness in a rotary evaporator at reduced pressure. The residue was muddled in a little acetone and collected by filtration. The filter cake was washed with acetone. The resulting precipitate was composed mainly of compound **16**. The stock solution was evaporated in a rotary evaporator at reduced pressure and the residue was subjected to preparative chromatography. Mixed chloroform, acetonitrile, heptane and acetic acid in a ratio of 6:1:0.5:0.1 *v/v* were used as the eluent. The fractions containing compounds **16** and **17** were collected, for which Rfs were 0.69 and 0.31, respectively. The solvents were removed separately from the fractions containing the compounds. The precipitate that had earlier been obtained containing compound **16** was combined with the residue obtained after removal of the solvents from the fraction with Rf = 0.69 and recrystallized twice from chloroform. The residue after removal of the solvents from the fraction with Rf = 0.31 was recrystallized twice from acetone.

The result was compound **16** as a white crystalline powder. Yield: 0.71 g (91.7% assay (HPLC)), 2.30 mmol (7.7% calculated as compound **2**). Mp = 174–176 °C (CH_3_C(O)CH_3_). IR (KBr): ν = 3300, 3061, 3013, 2968, 1713, 1693, 1600, 1583, 1508, 1468, 1454, 1404, 1390, 1318, 1291, 1234, 1180, 1115, 1071,1030, 936, 915, 833, 813, 802, 715, 706, 679, 642 cm^−1^. ^1^H NMR (DMSO-d6): δ = 5.39 (s, 2H), 7.53–7.60 (m, 4H), 7.64–7.73 (m, 2H), 8.03 (dd, J_1_ = 17.7 Hz, J_2_ = 7.4 Hz, 4H), 11.57 (s, 1H) ppm. ^13^C{1H} NMR (DMSO-d6): δ = 65.4, 129.0, 129.3, 129.7, 129.8, 132.7, 133.6, 134.1, 165.8, 167.3, 170.5 ppm. The structure of the compound was validated by X-ray diffraction (Sub. 2.2. and Supplementary Materials).

The result was compound **17** as a white crystalline powder. Yield: 0.14 g (98.2% assay (HPLC)), 0.49 mmol (1.6% calculated as compound **2**). Mp = 177–179 °C (CH_3_C(O)CH_3_). IR (KBr): ν = 3304, 3170, 3067, 2954, 1716, 1697, 1640, 1602, 1580, 1544, 1503, 1476, 1407, 1389, 1253, 1234, 1217, 1182, 1163, 1074, 1027, 990, 921, 800, 716, 693, 646 cm^−1^. ^1^H NMR (DMSO-d6): δ = 4.45 (d, J = 5.6 Hz, 2H), 7.48–7.58 (m, 5H), 7.65 (t, J = 7.2 Hz, 1H), 7.94 (dd, J_1_ = 23.7 Hz, J_2_ = 7.7 Hz, 4H), 8.81 (t, J = 5.2 Hz, 1H), 11.3 (s, 1H) ppm. ^13^C{1H} NMR (DMSO-d6): δ = 45.3, 127.7, 128.8, 128.9, 129.0, 131.9, 133.3, 134.3, 167.0, 167.1, 172.0 ppm. The structure of the compound was validated by X-ray diffraction (Sub. 2.2. and Supplementary Materials).

### 4.9. Synthesis of N-Formylbenzamide (***18***), N-(Tetrahydrofuran-2-yl)benzamide (***13***), and 1,2-Bis(benzoylamino)-1,2-ethanediol (***3***) (Isomer 2)

A mixture of **1** (10 g, 82.55 mmol), glyoxal (6 g, 40%, 41.35 mmol), THF (100 mL) and HCOOH (3 mL) was refluxed with vigorous stirring in a round-bottom flask equipped with a reflux condenser for 16 h. A half of the solvent was withdrawn from the reaction mixture in a rotary evaporator at reduced pressure, and the whole mixture was held at room temperature for 3 days. After that, the suspension was filtered and the filter cake was washed with THF. The stock solution after the filtration and THF after the residue washing were combined and evaporated to dryness at reduced pressure in a rotary evaporator. The remaining resin was subjected to preparative chromatography. Mixed chloroform, acetonitrile, heptane and acetic acid in a ratio of 6:1:0.5:0.1 by volume were used as the eluent. The fractions containing compounds **13** and **18** (Rf 0.48 and 0.24, respectively) were collected separately. The solvents of the collected fractions were evaporated separately to dryness at reduced pressure in a rotary evaporator. The resultant residues were recrystallized individually from mixed diethyl ether/chloroform at ratios of 5:1 and 1:1 by volume, respectively.

The eluent was further replaced by 1:1 *v/v* mixed chloroform/acetone, and the compounds left in the column (preparative chromatography) were eluted. The fractions were collected fractionally under HPLC control. The first to come out of the column was amide **1** followed by mixed compounds, among which was compound **3**. The fraction containing **3** was collected and evaporated to dryness in a rotary evaporator at reduced pressure. The residue was muddled in chloroform for 20 min, combined by filtration and washed with chloroform. The filter cake was extracted with a great excess of boiling acetone with stirring and then filtered. The procedure was repeated until all of compound **3** was extracted from the residue (HPLC). The extracts were combined and evaporated to dryness at reduced pressure in a rotary evaporator. The residue was extracted many-fold with a great volume of THF until most of compound **3** was extracted. The combined extracts were crystallized fractionally (slowly evaporating the solvent at room temperature). The precipitates composed of pure compound **3** (HPLC) were combined.

The result was compound **3** as a white crystalline powder. Yield: 0.31 g (96.4% assay (HPLC)), 0.99 mmol (2.4% calculated as compound **1**). Mp = 167–168 °C (dec.). IR (KBr): ν = 3294, 3060, 2928, 1645, 1603, 1581, 1525, 1490, 1397, 1351, 1288, 1221, 1151, 1080, 1041, 1001, 926, 883, 845, 803, 694, 634 cm^−1^. ^1^H NMR (DMSO-d6): δ = 5.51 (s, 2H), 6.03 (s, 2H), 7.45–7.55 (m, 6H), 7.84 (d, J = 7.5 Hz, 4H), 8.68 (d, J = 7.7 Hz, 4H) ppm. ^13^C{1H} NMR (DMSO-d6): δ = 75.0, 127.9, 128.7, 131.8, 135.1, 167.0 ppm. Elemental analysis, calcd (%) for C_16_H_16_N_2_O_4_ (300.31): C, 63.99; H, 5.37; N, 9.33; found: C, 63.03; H, 5.32; N, 9.21.

The result was compound **13** as a white crystalline powder. Yield: 0.20 g (95.2% assay (HPLC)), 1.00 mmol (1.2% calculated as compound **1**). Mp = 137–138 °C (CH_3_CN). IR (KBr): ν = 3313, 3057, 2971, 2882, 1643, 1600, 1578, 1531, 1488, 1449, 1356, 1313, 1295, 1262, 1195, 1138, 1124, 1061, 1041, 1025, 985, 956, 939, 920, 880, 804, 716, 695, 647 cm^−1^. ^1^H NMR (DMSO-d6): δ = 1.76–1.95 (m, 2H), 1.98–2.13 (m, 2H), 3.68–3.84 (m, 2H), 5.76 (q, J_1_ = 12.2 Hz, J_2_ = 6.9 Hz, 1H), 7.47 (t, J = 7.3 Hz, 2H), 7.54 (t, J = 7.1 Hz, 1H), 7.86 (d, J = 7.5 Hz, 2H), 8.79 (d, J = 8.0 Hz, 1H) ppm. ^13^C{1H} NMR (DMSO-d6): δ = 25.0, 30.9, 66.8, 80.9, 127.9, 128.7, 131.8, 134.7, 166.9 ppm. The structure of the compound was validated by X-ray diffraction (Sub. 2.2. and Supplementary Materials).

The result was compound **18** as a white crystalline powder. Yield: 0.26 g (98.0% assay (HPLC)), 1.71 mmol (2.1% calculated as compound **1**). Mp = 103–104 °C (diethyl ether: CHCl_3_, 5: 1). IR (KBr): ν = 3272, 3172, 3070, 2928, 1726, 1691, 1672, 1599, 1581, 1503, 1461, 1365, 1352, 1250, 1239, 1210, 1157, 1077, 1061, 1029, 1001, 933, 886, 804, 748, 701, 674 cm^−1^. ^1^H NMR (DMSO-d6): δ = 7.55 (t, J = 7.4 Hz, 2H), 7.68 (t, J = 7.3 Hz, 1H), 8.02 (d, J = 7.8 Hz, 2H), 9.27 (s, 1H), 11.74 (s, 1H) ppm. ^13^C{1H} NMR (DMSO-d6): δ = 128.9, 129.1, 132.0, 133.9, 164.9, 168.0 ppm. The structure of the compound was validated by X-ray diffraction (Sub. 2.2. and Supplementary Materials).

### 4.10. Synthesis of N-(2-Phenyl-1,3-oxazol-5-yl)benzamide (***19***)

A mixture of diol **2** (3 g, 10 mmol) and TFA (75 mL) was refluxed with stirring for 4 h. Upon completion, the whole was evaporated to dryness in a rotary evaporator at reduced pressure. The residue was diluted with water and extracted with ethyl acetate. The extract was washed twice with water, then dried over Na_2_SO_4_, and evaporated to dryness in a rotary evaporator at reduced pressure. The residue after solvent removal was subjected to preparative chromatography. Mixed chloroform/acetonitrile in a ratio of 6:1 *v/v* were used as the eluent. The solvents were evacuated from the collected fraction with Rf = 0.56 in a rotary evaporator at reduced pressure. The residue after solvent removal was recrystallized twice from acetone to give compound **19** as a white crystalline powder.

Yield: 0.30 g (96.0% assay (HPLC)), 1.09 mmol (10.9% calculated as compound **2**). Mp = 176–178 °C (CH_3_C(O)CH_3_). IR (KBr): ν = 2956, 2925, 2853, 2787, 1685, 1616, 1599, 1581, 1561, 1547, 1486, 1447, 1340, 1307, 1282, 1251, 1110, 1067, 1022, 1005, 887, 804, 774, 714, 706, 691, 654 cm^−1^. ^1^H NMR (DMSO-d6): δ = 7.27 (s, 1H), 7.49–7.58 (m, 5H), 7.64 (t, J = 7.2 Hz, 1H), 7.99 (dd, J_1_ = 39.0 Hz, J_2_ = 7.2 Hz, 4H), 11.71 (s, 1H) ppm. ^13^C{1H} NMR (DMSO-d6): δ = 114.1, 125.7, 127.3, 128.4, 129.1, 129.7, 130.5, 132.8, 133.0, 146.5, 154.4, 164.4 ppm. The structure of the compound was validated by X-ray diffraction (Sub. 2.2. and Supplementary Materials).

## 5. Conclusions

Here, we investigated in detail an acid-catalyzed condensation between benzamide and glyoxal in a molar ratio of 2:1 in polar protic and aprotic solvents. Under the conditions used, benzamide was low-activity over a wide range of acidities and temperatures and reacted slowly with glyoxal, in which case it underwent hydrolysis to benzoic acid. At the same time, glyoxal was involved in cascade side reactions and was removed from the reaction mixture. In most cases, the condensation ended before benzamide was exhausted. The general picture showed that similar processes were observed to take place in polar aprotic and protic solvents, which resulted in a great number of compounds. In the course of the study, we isolated and identified 16 compounds. Of these, seven condensation products of benzamide and glyoxal and 1 reaction product of benzamide and glyoxal in THF were first synthesized in this study. In particular, a geminal diol, *N*,*N*’-(2,2-dihydroxyethane-1,1-diyl)dibenzamide, was synthesized and identified. Two isomeric 1,2-bis(benzoylamino)-1,2-ethanediols were isolated and identified. *N*,*N*’-(1-oxoethane-1,2-diyl)dibenzamide and 2-oxo-2-[(phenylcarbonyl)amino]ethyl benzoate were synthesized that appeared to ensue from the 1,2-hydride shift. *N*-polysubstituted 1,4-dioxane-2,3,5,6-tetramine was obtained for the first time, whose structure can be of interest as a scaffold for novel explosives. This compound features a high melting point and a low solubility. It was discovered that DMSO, THF and HCOOH could engage in a reaction with benzamide or condensation products thereof and glyoxal under acid-catalyzed conditions. This aspect should be borne in mind when exploring the condensation between ammonia derivatives and glyoxal in those solvents. The formation of the diimine by the condensation between benzamide and glyoxal or by transformation of 1,2-bis(benzoylamino)-1,2-ethanediol was not established.

This study allows for the conclusion that the main factors impeding the acid-catalyzed cascade condensation of benzamide (probably all carbamides) with glyoxal to polyheterocyclic caged compounds are the low resistance of the carbamide group to hydrolysis and the tendency of the condensation products towards intramolecular transpositions.

## Figures and Tables

**Table 1:** Condensation products of benzamide with glyoxal in HCl of varied concentrations.

Entry	ω(HCl ^1^), % ^2^/ω(H_2_O), % ^2^	Composition of Principal Reaction Products (HPLC), %
1	23.12/67.24	Residue (0.057 g) ^3^: 1 (2.6), 2 (75.1), 4 (1.2), 5 (0.06), 6 and 8 (3.6) Filtrate: 1 (75.3), 2 (1.1), 3 (2.5), 4 (0.5), 6 and 8 (3.8), 7 (5.8)
2	20.09/70.12	Residue (0.451 g) ^3^: 1 (1.2), 2 (74.6), 4 (4.1), 5 (0.04), 6 and 8 (8.3), 7 (10.5) Filtrate: 1 (81.9), 2 (0.2), 3 (0.5), 6 and 8 (2.0), 7 (6.37)
3	16.98/73.09	Residue (0.433 g) ^3^: 1 (1.8), 2 (82.4), 4 (2.5), 6 and 8 (6.5), 7 (6.6) Filtrate: 1 (84.6), 7 (6.4)

Note: ^1^ In the pure form. ^2^ Mass content in the mixture. ^3^ Compound **1** was taken as 1 g.

**Table 2:** Acid-catalyzed condensation products of benzamide and glyoxal in polar aprotic solvents.

Entry	ω(Solvent), % ^1^/ ω(Acid), % ^1^/ ω(H_2_O), %^1^	Composition of Principal Reaction Products (HPLC), %
1	78.88 (CH_3_C(O)CH_3_)/ 4.55 (PTSA)/ 4.09 (H_2_O)	residue (0.138 g) ^2^: 4 (8.7), 5 (61.5), 12 (17.6); filtrate: 1 (35.5), 2 (2.1), 3 (1.6), 4 (5.4), 5 (1.0), 6 and 8 (5.1), 7 (22.3), 12 (0.6)
2	78.92 (CH_3_CN)/ 4.54 (PTSA)/ 4.08 (H_2_O)	residue (0.058 g) ^2^: 1 (0.6), 4 (10.0), 5 (34.3), 12 (40.4); filtrate: 1 (30.7), 2 (1.0), 4 (6.4), 5 (0.1), 6 and 8 (9.3), 7 (26.5), 12 (0.7)
3	83.98 (DMSO)/ 3.46 (PTSA)/ 3.11 (H2O)	1 (61.1), 2 (6.4), 3 (5.6), 4 (0.6), 6 and 8 (1.1), 7 (1.3), 12 (0.04)
4	80.90 (THF)/ 4.12 (PTSA)/ 3.70 (H_2_O)	residue (0.157 g) ^2^: 5 (55.4); filtrate: 1 (33.0), 2 (0.8), 3 (1.6), 4 (3.0), 5 (0.9), 6 and 8 (3.4), 7 (21.2), 12 (0.7), 13 (5.1)
5	76.99 (CH_3_C(O)CH_3_)/ 7.31 (TFA)/ 3.53 (H_2_O)	residue (0.142 g) ^2^: 2 (99.0), 5 (1.0); filtrate: 1 (59.8), 2 (1.6), 3 (3.3), 4 (1.6), 5 (0.1), 6 and 8 (1.9), 7 (3.0), 12 (0.4)
6	77.03 (CH_3_CN)/ 7.30 (TFA)/ 3.52 (H_2_O)	residue (0.082 g) ^2^: 1 (0.7), 2 (84.4), 4 (0.8), 5 (13.3); filtrate: 1 (48.7), 2 (1.9), 3 (3.1), 4 (4.9), 5 (0.7), 6 and 8 (3.9), 7 (4.6), 12 (0.7)
7	82.44 (DMSO)/ 5.58 (TFA)/ 2.69 (H_2_O)	1 (69.4), 2 (5.0), 3 (5.5), 4 (0.1), 6 and 8 (0.6), 7 (0.6), 15 (2.0)
8	79.14 (THF)/ 6.63 (TFA)/ 3.20 (H_2_O)	residue (0.028 g) ^2^: 1 (6.1), 2 (81.0), 5 (4.9); filtrate: 1 (54.0), 2 (4.2), 3 (5.3), 4 (1.0), 6 and 8 (1.3), 7 (3.3), 12 (0.03), 13 (5.0)

Note: ^1^ Mass content in the mixture. ^2^ Compound **1** was taken as 1 g.

**Table 3:** Transformation products of diol **2** in acidified polar aprotic solvents or organic acids.

Entry	ω(Solvent), % ^1^/ ω(Acid), % ^1^	T °C/ t, h	Composition of Principal Reaction Products (HPLC), %
1	97.6 (HCOOH)	B.P./7	**1** (54.1), **7** (16.0), **16** (12.2), **17** (2.8), **18** (8.0)
2	98 (TFA)	B.P./7	**1** (24.8), **2** (0,5), **3** (1.6), **4** (2.1), **7** (26.6), **16** (14.0), **17** (3.0), **19** (21.7)
3	97.2 (AcOH)	B.P./7	**1** (75.0), **7** (13.3), **15** (0.3), **17** (5.3)
4	92,9 (CH_3_CN)/ 3,5 (TFA)	B.P./7	**1** (28.8), **2** (10.8), **4** (2.2), **12** (4.5), **17** (10.1), **16** (9.7)
5	94,8 (DMSO)/ 2,6 (TFA)	115/7	**1** (58.8), **2** (0.4), **7** (19.4), **15** (12.6), **17** (4.4)
6	95.3 (DMSO)/ 0.7 (HCl ^2^)	115/7	**1** (9.5), **7** (12.2), **15** (52.2)
7	94.3 (DMSO)/ 3.1 (H_2_SO_4_)	115/7	**1** (25.9), **7** (22.3), **15** (42.3), **17** (0.3)

Note: ^1^ Mass content in the mixture. ^2^ In the pure form.

**Table 4:** H-bonding parameters for compounds **6**, **8**, **13** and **16–19**.

Comp.	H-Bonding	D-H, Å	H…A, Å	D…A, Å	D-H…A,°
**6**	O4-H…O3	0.82	2.04	2.802(3)	154
	O3-H…O4	0.85(3)	1.94(3)	2.762(3)	164(3)
	N1-H…O1	0.85(3)	2.11(3)	2.941(3)	164(3)
	N2-H…O2	0.83(3)	2.13(3)	2.920(3)	160(3)
**8**	N1′-H…O1	0.98(4)	2.09(4)	3.046(5)	165(4)
	N2-H…O2′	0.90(4)	2.08(4)	2.874(5)	147(4)
	N2′-H…O2	0.86(3)	2.10(3)	2.910(4)	156(3)
	N1-H…O1′	0.80(3)	2.25(3)	3.026(5)	167(3)
**13**	N1-H…O1	0.91(2)	2.03(2)	2.913(2)	162(2)
**16**	N1-H…O2	0.85(2)	2.15(2)	2.973(2)	162(2)
**17**	N1-H…O2	0.83(2)	2.19(2)	2.991(2)	161(2)
	N2-H…O3	0.84(2)	2.12(2)	2.919(2)	160(2)
**18**	N1-H…O2	0.88(2)	2.14(2)	3.007(2)	170(2)
**19**	N1-H…N2	0.89(1)	2.05(1)	2.931(1)	169(1)

## Data Availability

Not applicable.

## Supplementary Materials

NMR spectra for all the compounds and X-ray diffraction data for new compounds are available.

## Sample Availability

Samples of the compounds are not available from the authors.
