# Supplementary material for: Acid-Catalyzed Condensation of Benzamide with Glyoxal, and Reaction Features
Source: Molecules. 2022 Feb 7;27(3):1094. doi: 10.3390/molecules27031094 (PMC8838861; doi:10.3390/molecules27031094)
Supplement: Supplementary file 1 [file molecules-27-01094-s001.zip › molecules-1516077-supplementary.pdf]

Supplementary Materials

Acid-Catalyzed Condensation of Benzamide with Glyoxal, and Reaction Features

Artyom E. Paromov,*[1] Irina A. Shchurova,[1] Alla I. Rogova,[1] Irina Yu. Bagryanskaya,[2] and Dmitriy N. Polovyanenko[2]

[1] *Laboratory for Chemistry of Nitrogen Compounds, Institute for Problems of Chemical and Energetic Technologies, Siberian Branch of the Russian Academy of Sciences (IPCET SB RAS), Biysk 659322, Russia*

[2] *Department of Chemistry, Novosibirsk Institute of Organic Chemistry, Siberian Branch of the Russian Academy of Sciences, Novosibirsk 630090, Russia*

Table of Contents

^{1}H and ^{13}C NMR of compound **2** S2

^{1}H and ^{13}C NMR of compound **3** S4

^{1}H and ^{13}C NMR of compound **4** S6

^{1}H and ^{13}C NMR of compound **5** S8

X-ray diffraction data for compound **6** S10

X-ray diffraction data for compound **8** S11

^{1}H and ^{13}C NMR of compound **9** S12

^{1}H and ^{13}C NMR of compound **10** S14

^{1}H and ^{13}C NMR of compound **11** S16

^{1}H and ^{13}C NMR of compound **12** S18

^{1}H and ^{13}C NMR of compound **13** S20

X-ray diffraction data for compound **13** S22

^{1}H and ^{13}C NMR of compound **15** S23

^{1}H and ^{13}C NMR of compound **16** S25

X-ray diffraction data for compound **16** S27

^{1}H and ^{13}C NMR of compound **17** S28

X-ray diffraction data for compound **17** S30

^{1}H and ^{13}C NMR of compound **18** S31

X-ray diffraction data for compound **18** S33

^{1}H and ^{13}C NMR of compound **19** S34

X-ray diffraction data for compound **19** S36

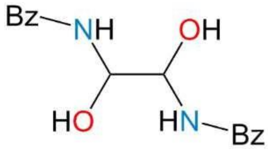

1,2-Bis(benzoylamino)-1,2-ethanediol (**2**) (isomer 1)

^{1}H (400 MHz) NMR (DMSO-d6, 24°C)

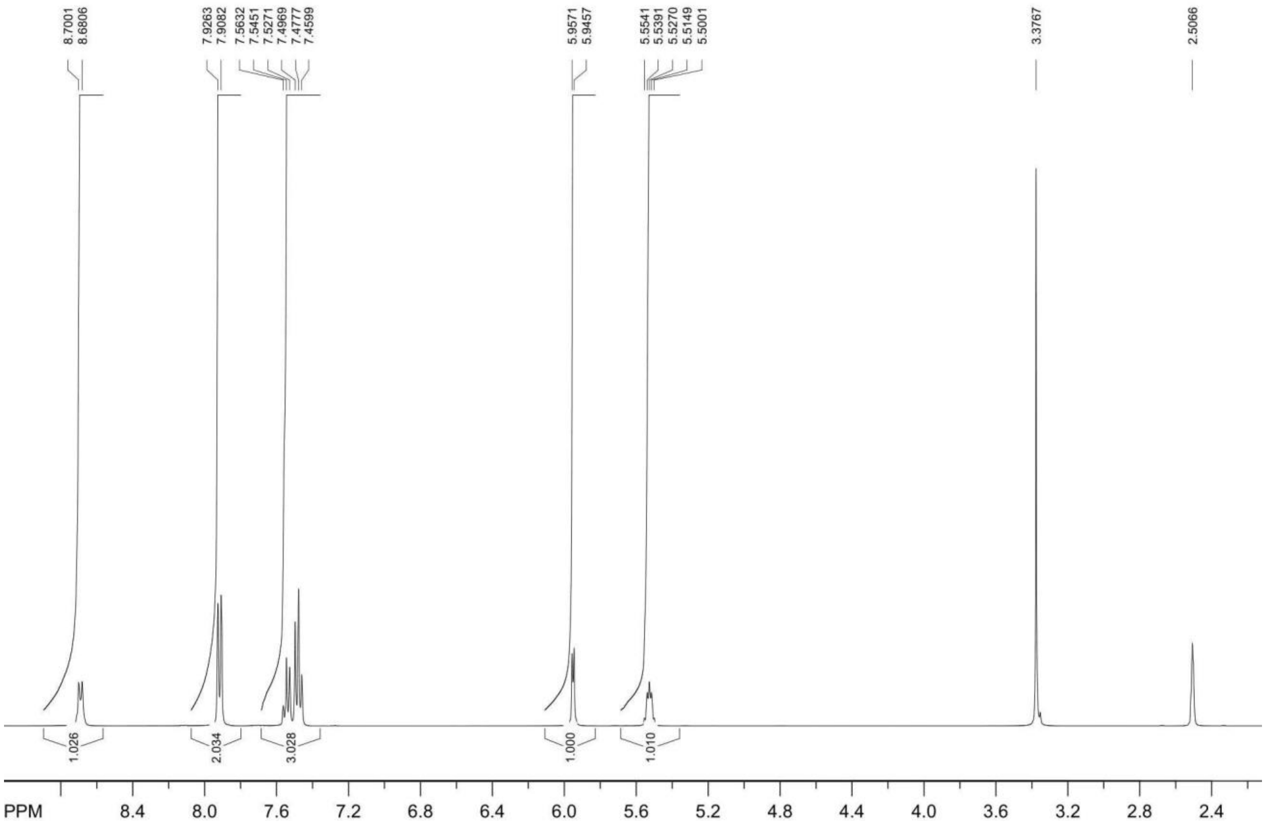

^{13}C (100 MHz) NMR (DMSO-d6, 24°C)

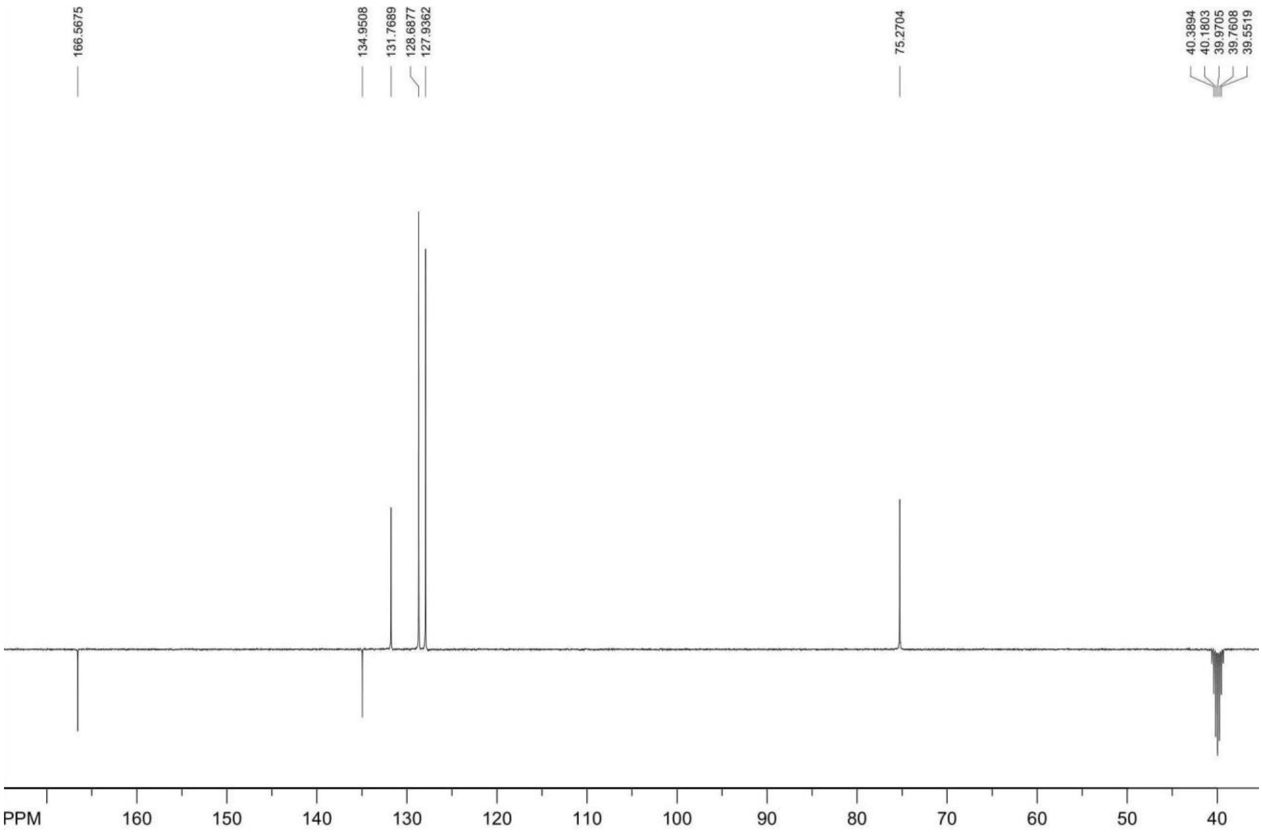

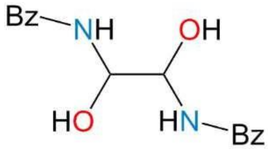

1,2-Bis(benzoylamino)-1,2-ethanediol (**3**) (isomer 2)

^{1}H (400 MHz) NMR (DMSO-d6, 24°C)

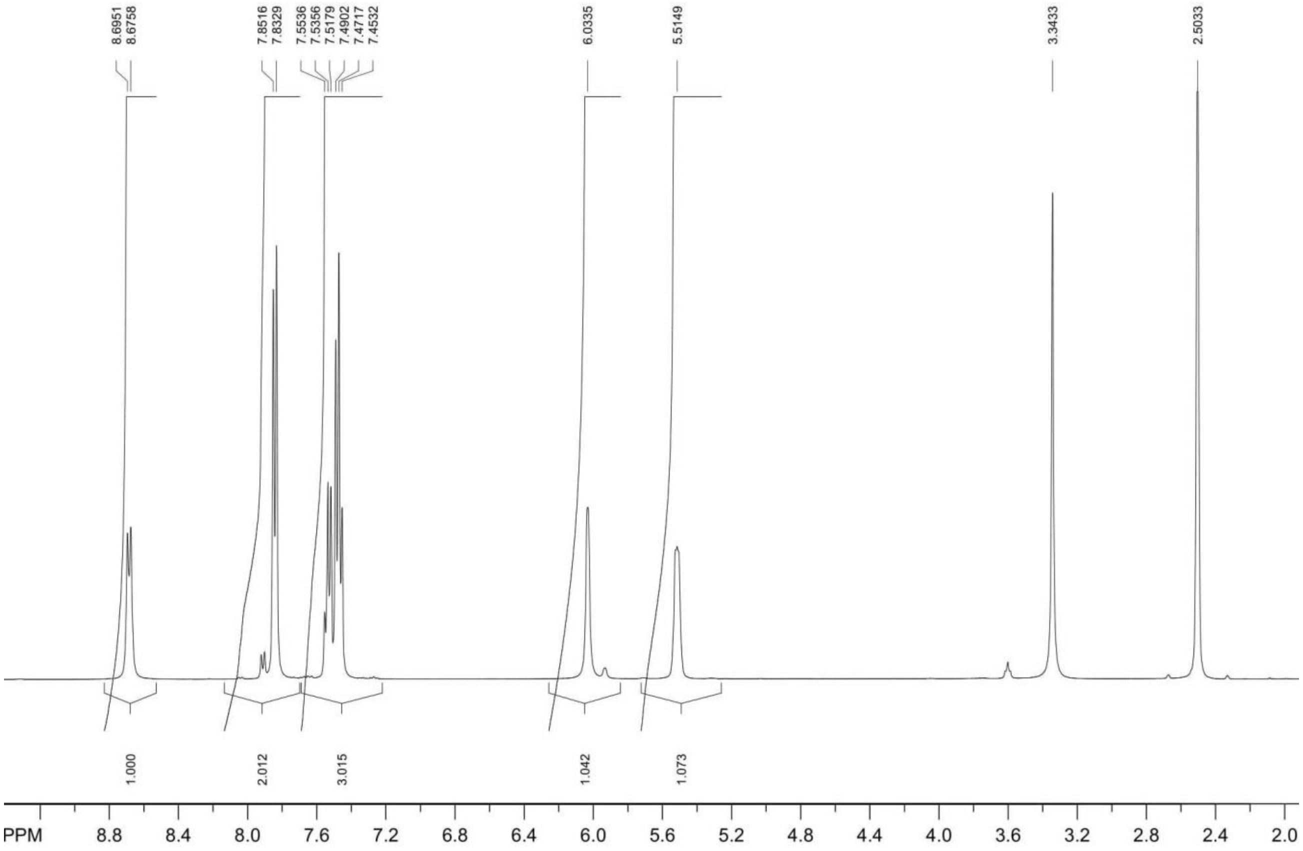

^{13}C (100 MHz) NMR (DMSO-d6, 24°C)

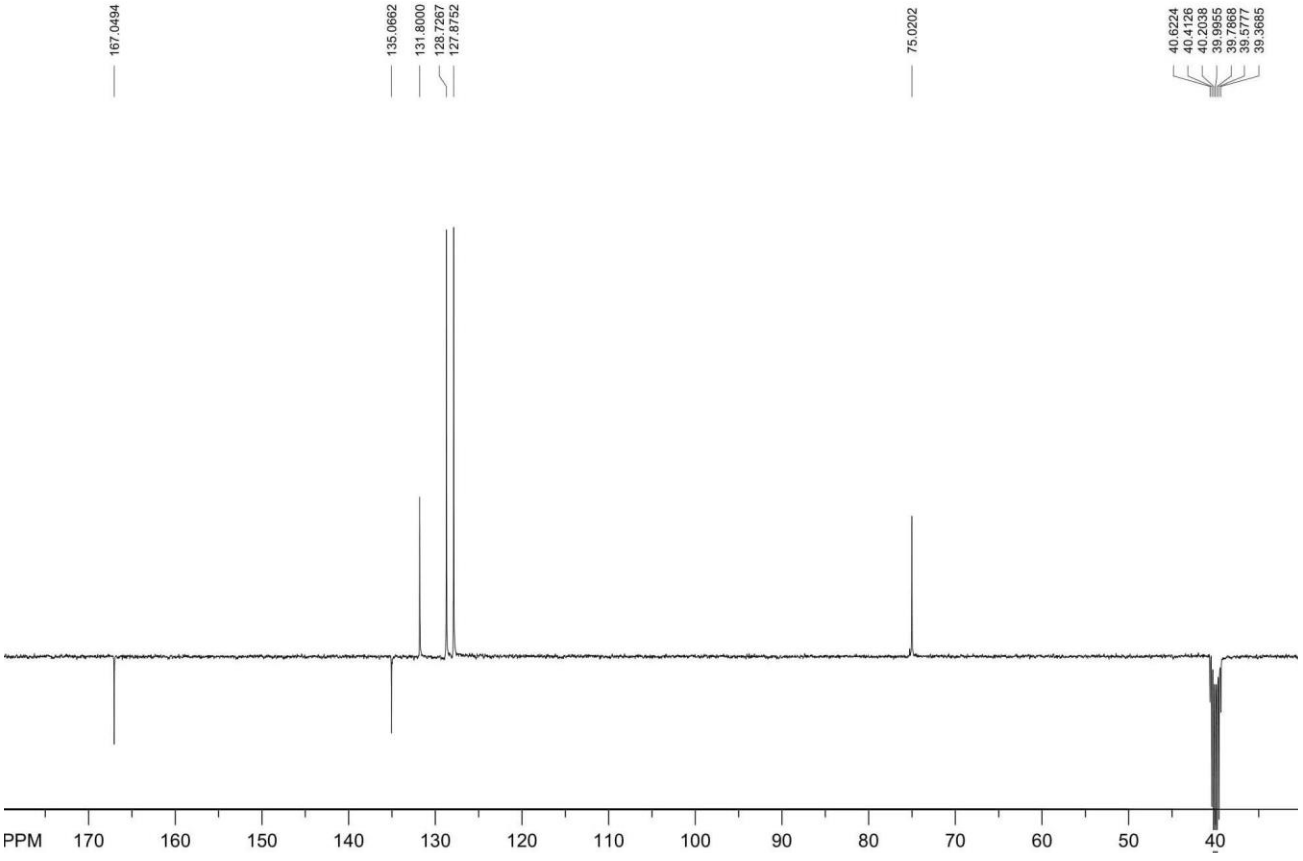

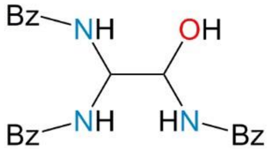

N,N',N"-(2-Hydroxyethane-1,1,2-triyl)tribenzamide (**4**)

^{1}H (400 MHz) NMR (DMSO-d6, 24ºC)

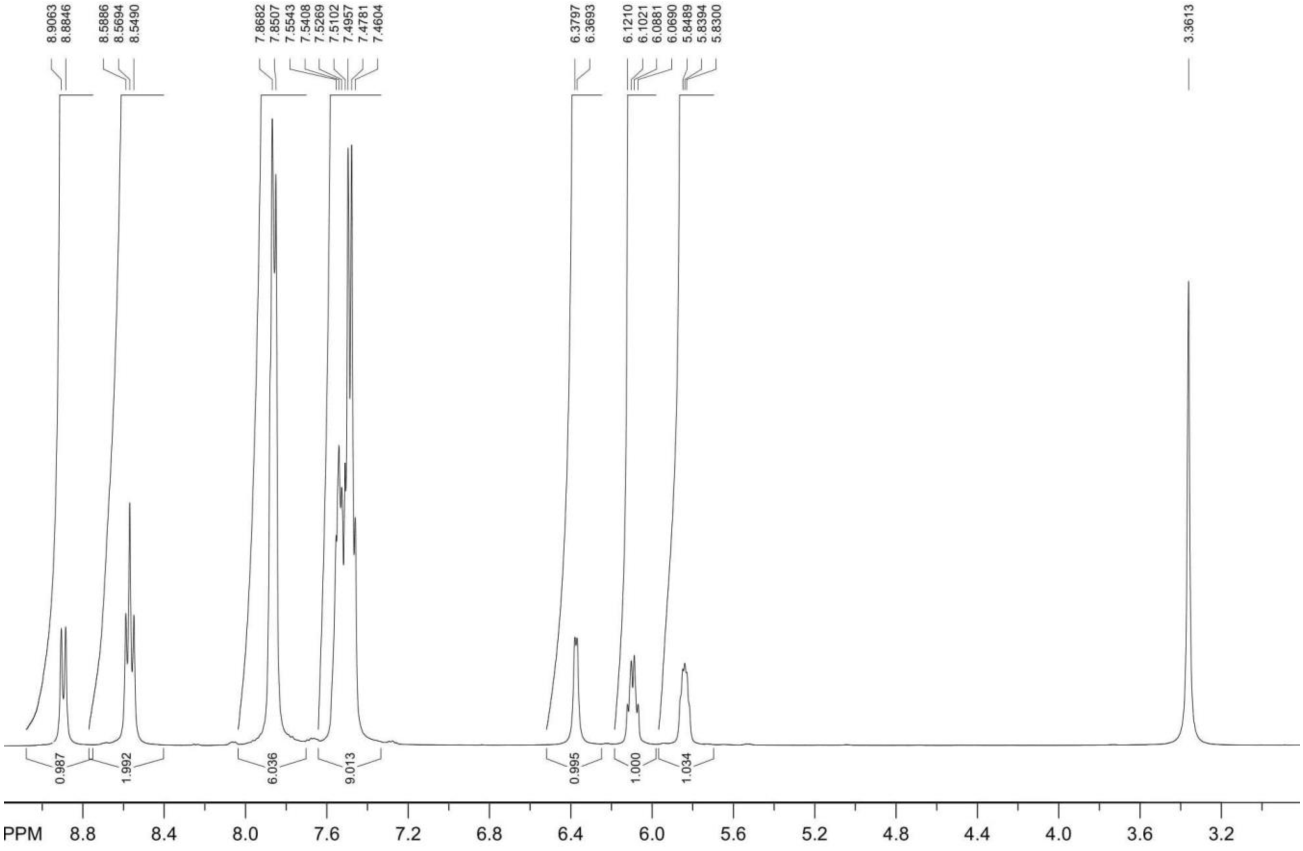

^{13}C (100 MHz) NMR (DMSO-d6, 24ºC)

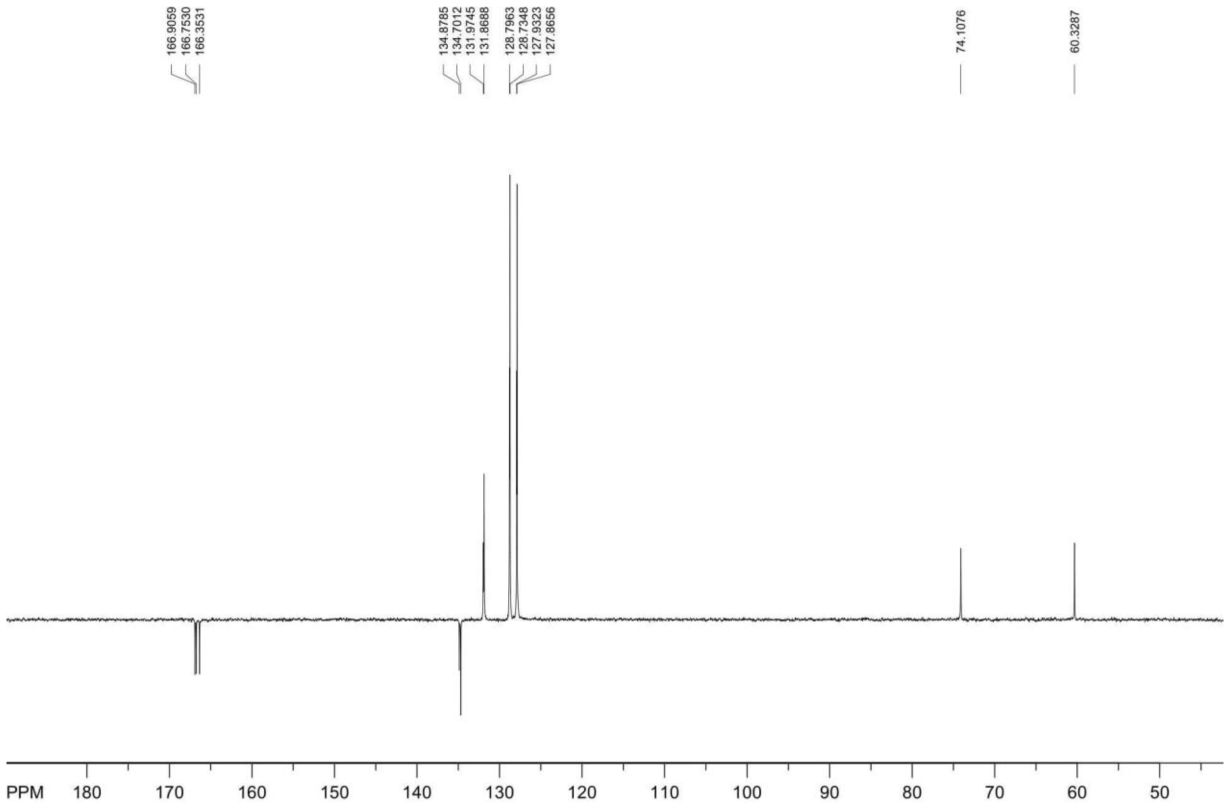

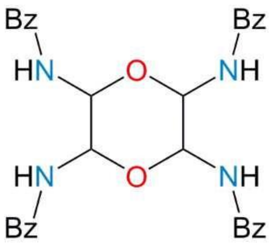

N,N',N'',N'''-(1,4-Dioxane-2,3,5,6-tetrayl)tetrabenzamide (**5**)

^{1}H (400 MHz) NMR (DMSO-d6, 24ºC)

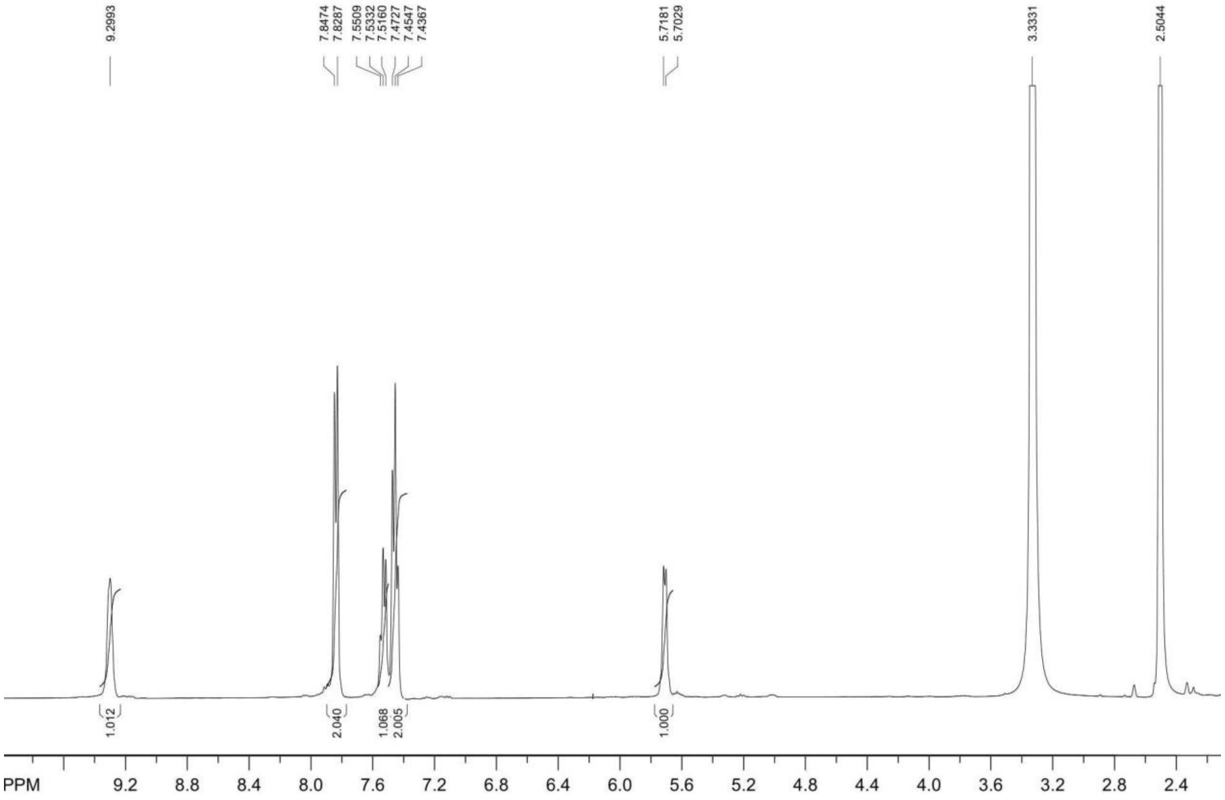

^{13}C (100 MHz) NMR (DMSO-d6, 24ºC)

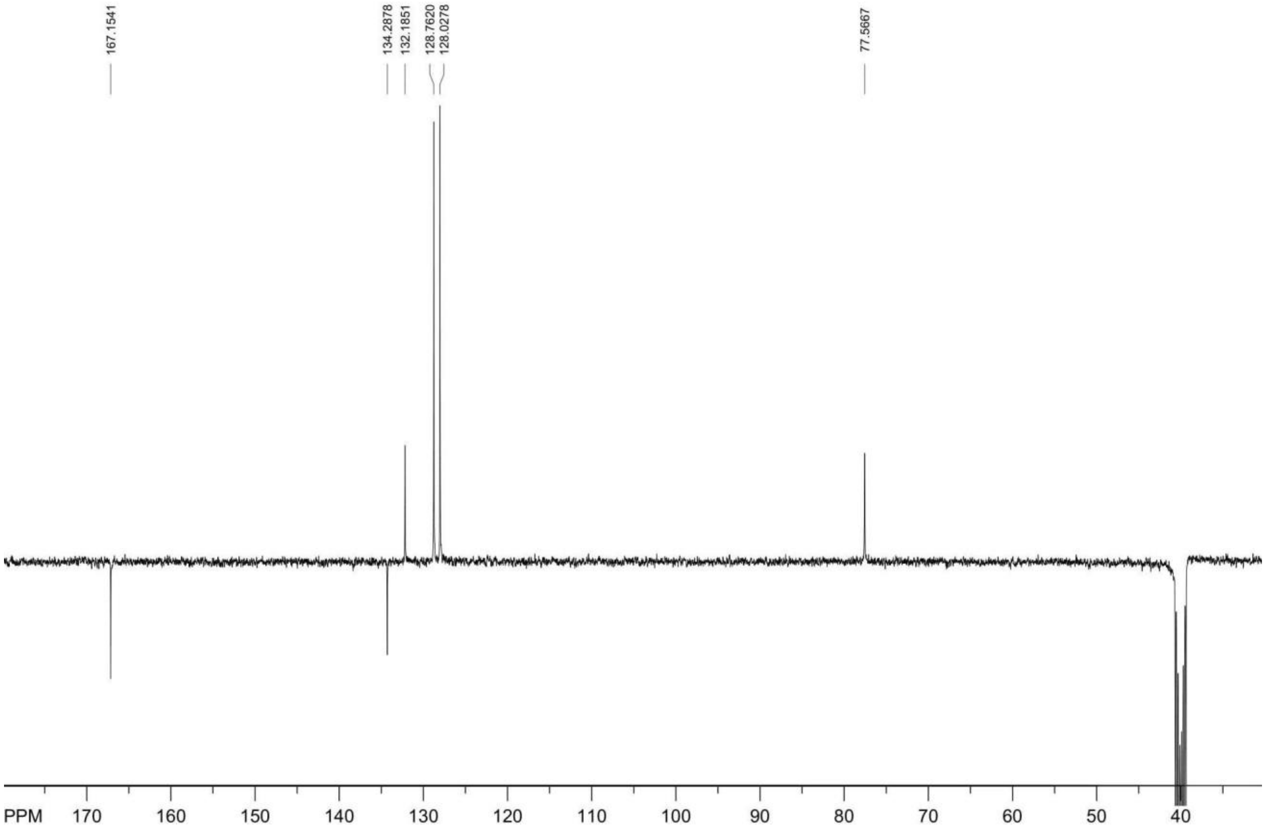

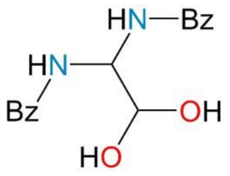

X-ray diffraction data for N,N'-(2,2-dihydroxyethane-1,1-diyl)dibenzamide (**6**)

Table 1 XRD data for compound **6**.

Empirical formula	$C_{16}H_{16}N_2O_4$
Formula weight	300.31
Crystal system	Monoclinic
Space group	$P2_1/c$
Unit cell dimensions *a* Å	13.412(2)
b Å	4.9999(5)
c Å	22.378(3)
α °	90
β °	94.604(4)
γ °	90
Volume Å^3	1495.8(3)
Z	4
Density (calcd.) Mg.m^{-3}	1.334
Abs. coefficient mm^{-1}	0.097
F(000)	632
Crystal size mm^3	0.02 x 0.04 x 0.85
Θ range for data collection °	2.3 – 25.1
Index ranges	$-15 \leq h \leq 15$, $-5 \leq k \leq 5$,
	$-26 \leq l \leq 26$
Reflections collected	16349
Independent reflections	2630 R(int)= 0.087
Completeness to θ %	99.9
Data / restraints / parameters	1480/ 3 / 133
Goodness-of-fit on F^2	1.11
Final R indices $I > 2\sigma(I)$	R_1 = 0.0581, wR_2 = 0.1392
Final R indices (all data)	R_1 = 0.1008, wR_2 = 0.1584
Largest diff. peak / hole e.Å^{-3}	0.20/ -0.22
CCDC	2108741

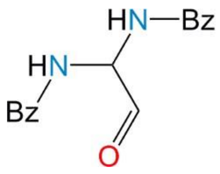

X-ray diffraction data for N,N'-(2-oxoethane-1,1-diyl)dibenzamide (**8**)

Table 2 XRD data for compound **8**.

Empirical formula	$C_{16}H_{14}N_2O_3$
Formula weight	282.29
Crystal system	Triclinic
Space group	P -1
Unit cell dimensions *a* Å	9.469(5)
b Å	10.165(6)
c Å	15.524(7)
α º	80.06(2)
β º	73.55(2)
γ º	78.34(2)
Volume Å^3	1392.9(13)
Z	4
Density (calcd.) Mg.m^{-3}	1.346
Abs. coefficient mm^{-1}	0.095
F(000)	592
Crystal size mm^3	0.01 x 0.4 x 0.30
Θ range for data collection °	2.1 – 25.0
Index ranges	$-11 \leq h \leq 11$, $-12 \leq k \leq 12$, $-18 \leq l \leq 18$
Reflections collected	17069
Independent reflections	4920 R(int) = 0.102
Completeness to θ %	99.9
Data / restraints / parameters	4920 / 0 / 395
Goodness-of-fit on F^2	1.01
Final R indices $I > 2\sigma(I)$	R_1 = 0.0606, wR_2 = 0.1088
Final R indices (all data)	R_1 = 0.1878, wR_2 = 0.1393
Largest diff. peak / hole e.Å^{-3}	0.19, -0.19
CCDC	2108742

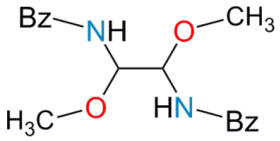

N,N'-(1,2-Dimethoxyethane-1,2-diyl)dibenzamide (**9**)

^{1}H (400 MHz) NMR (DMSO-d6, 24ºC)

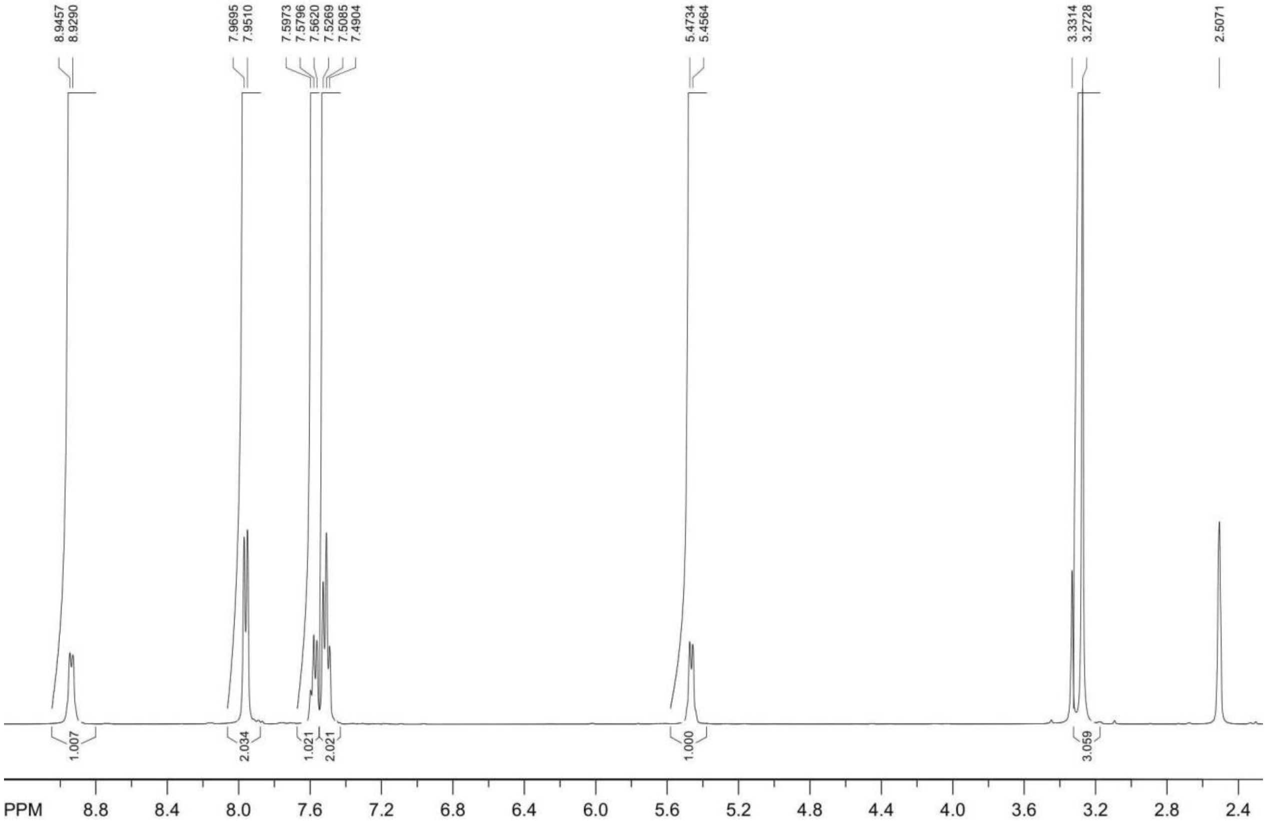

^{13}C (100 MHz) NMR (DMSO-d6, 24ºC)

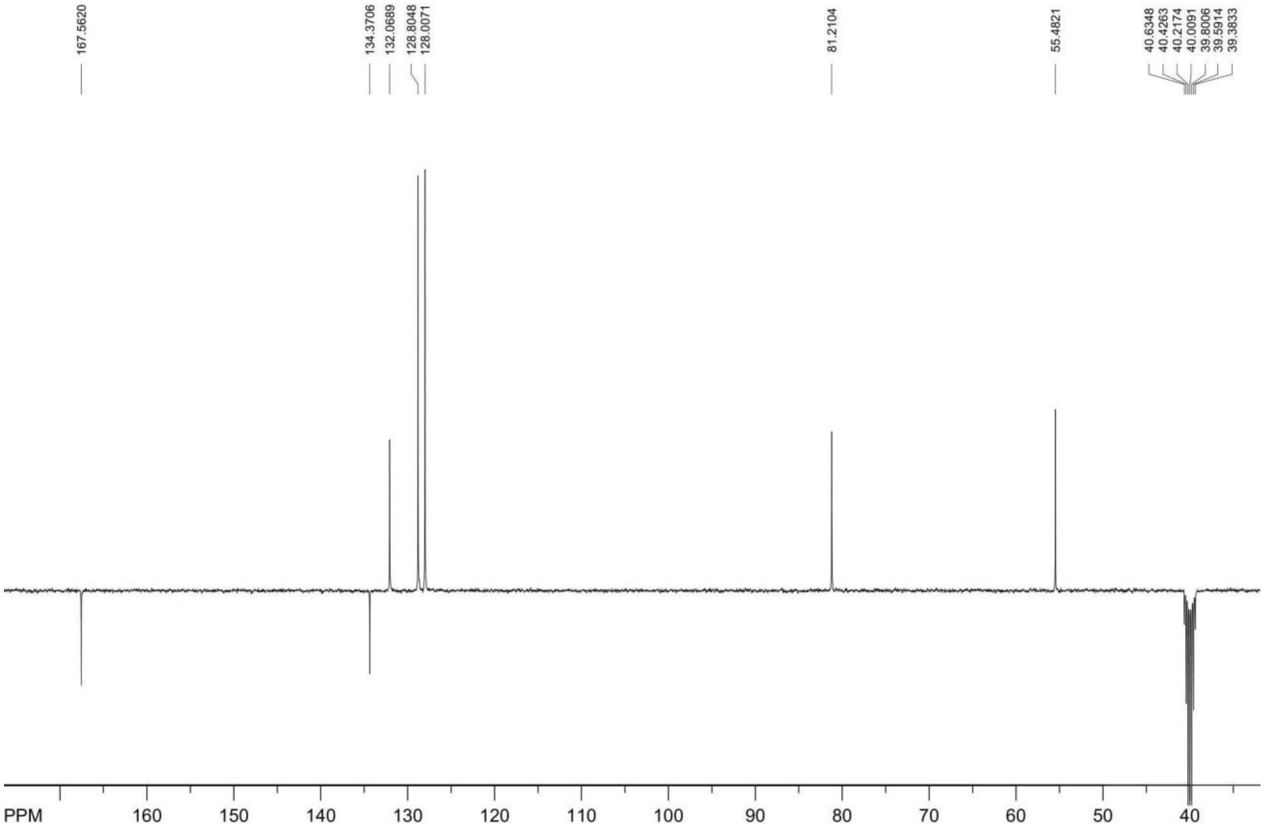

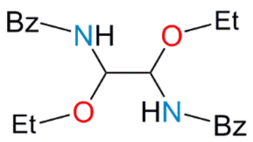

N,N'-(1,2-Diethoxyethane-1,2-diyl)dibenzamide (**10**)

^{1}H (400 MHz) NMR (DMSO-d6, 24ºC)

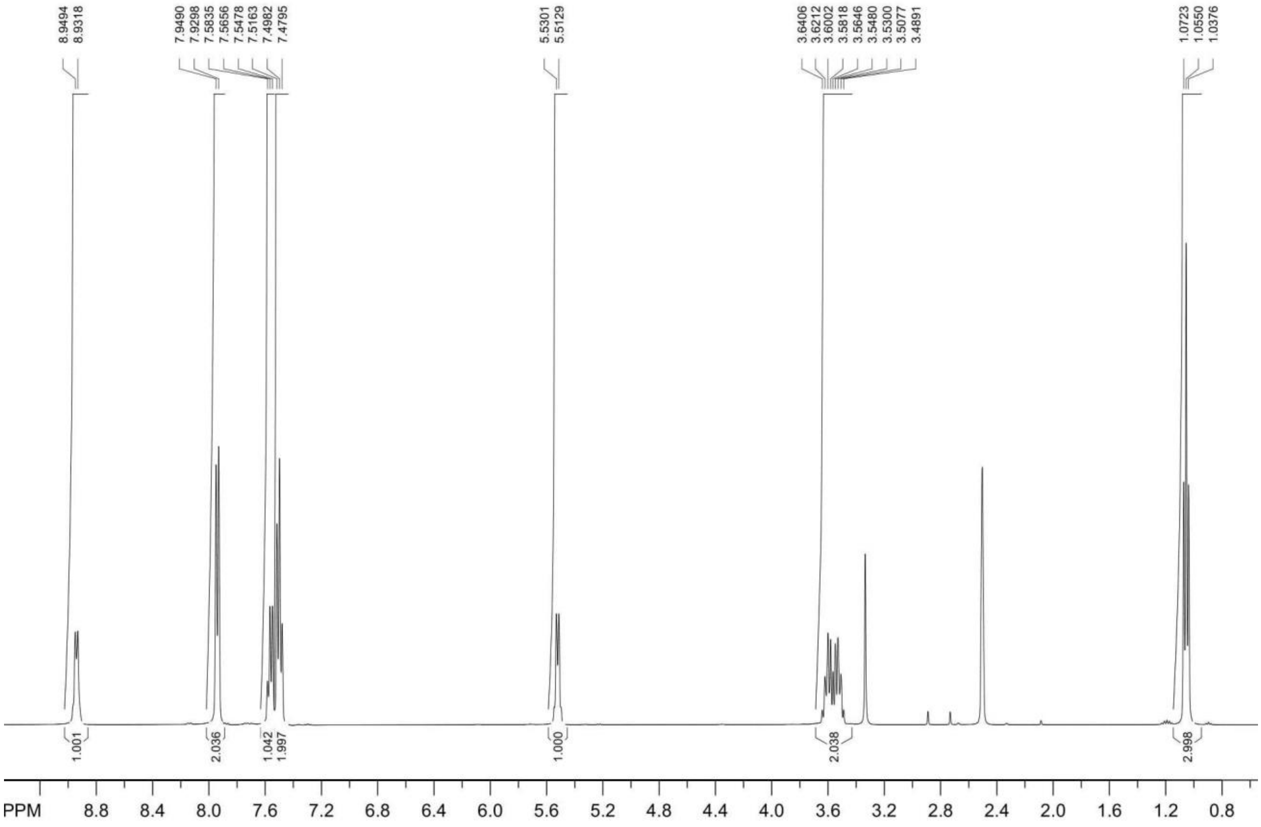

^{13}C (100 MHz) NMR (DMSO-d6, 24°C)

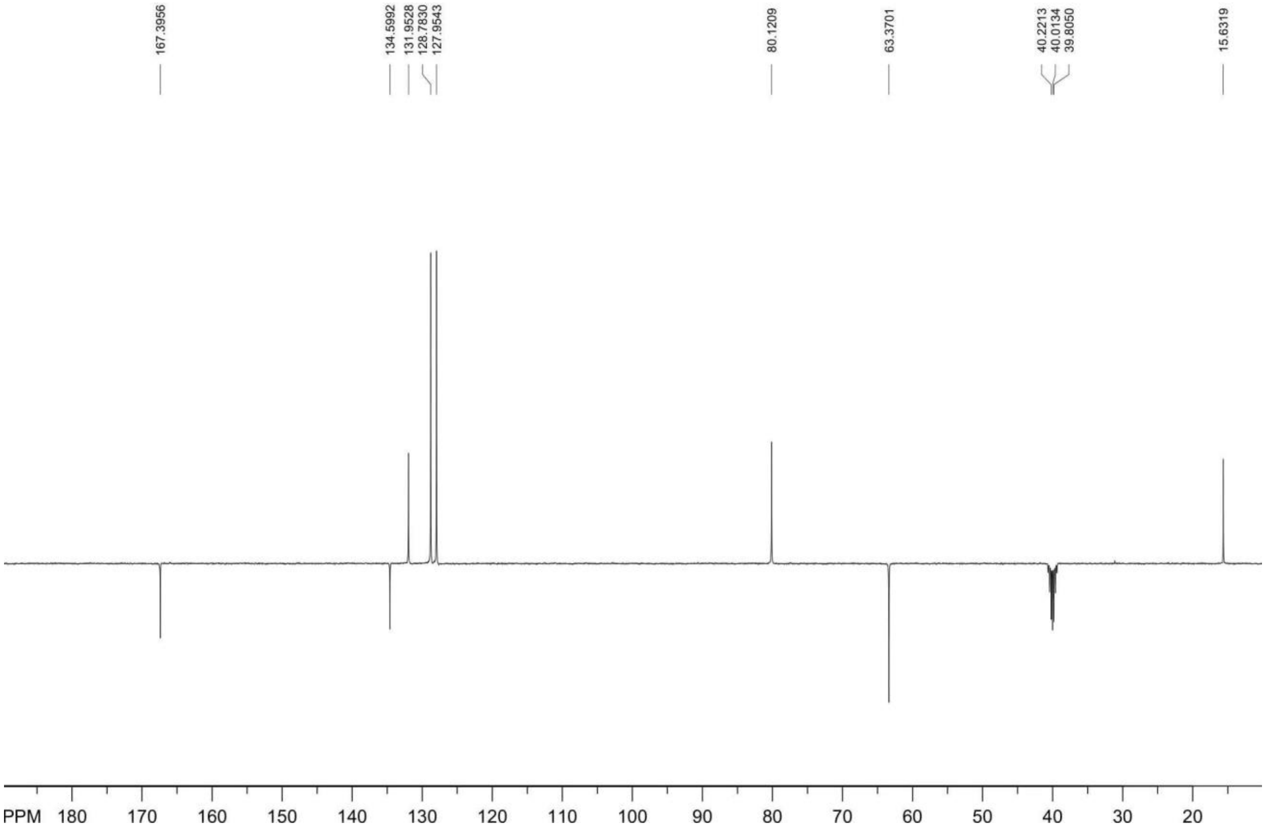

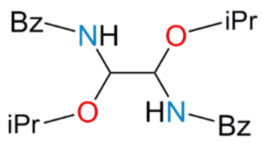

N,N'-(1,2-Diisopropoxyethane-1,2-diyl)dibenzamide (**11**)

^{1}H (400 MHz) NMR (DMSO-d6, 24ºC)

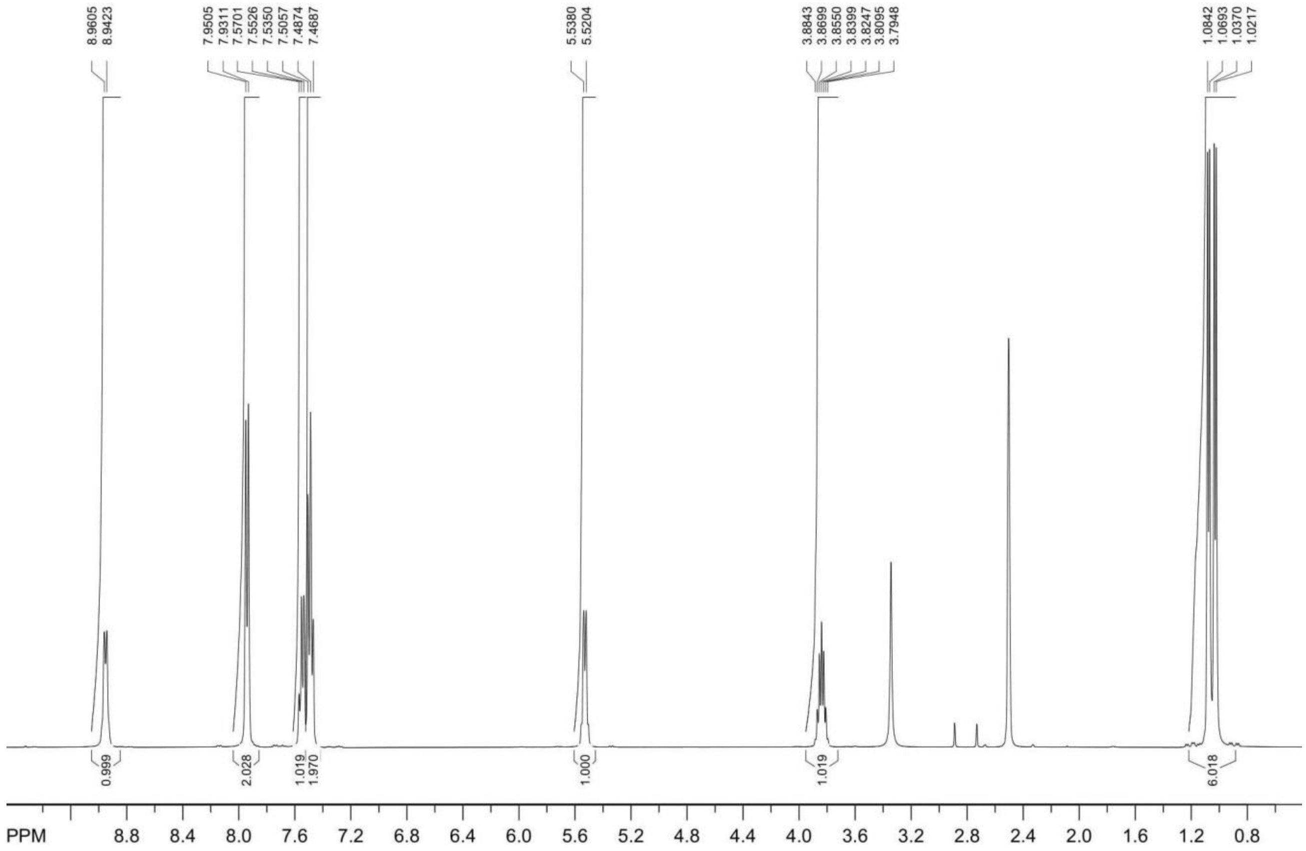

^{13}C (100 MHz) NMR (DMSO-d6, 24ºC)

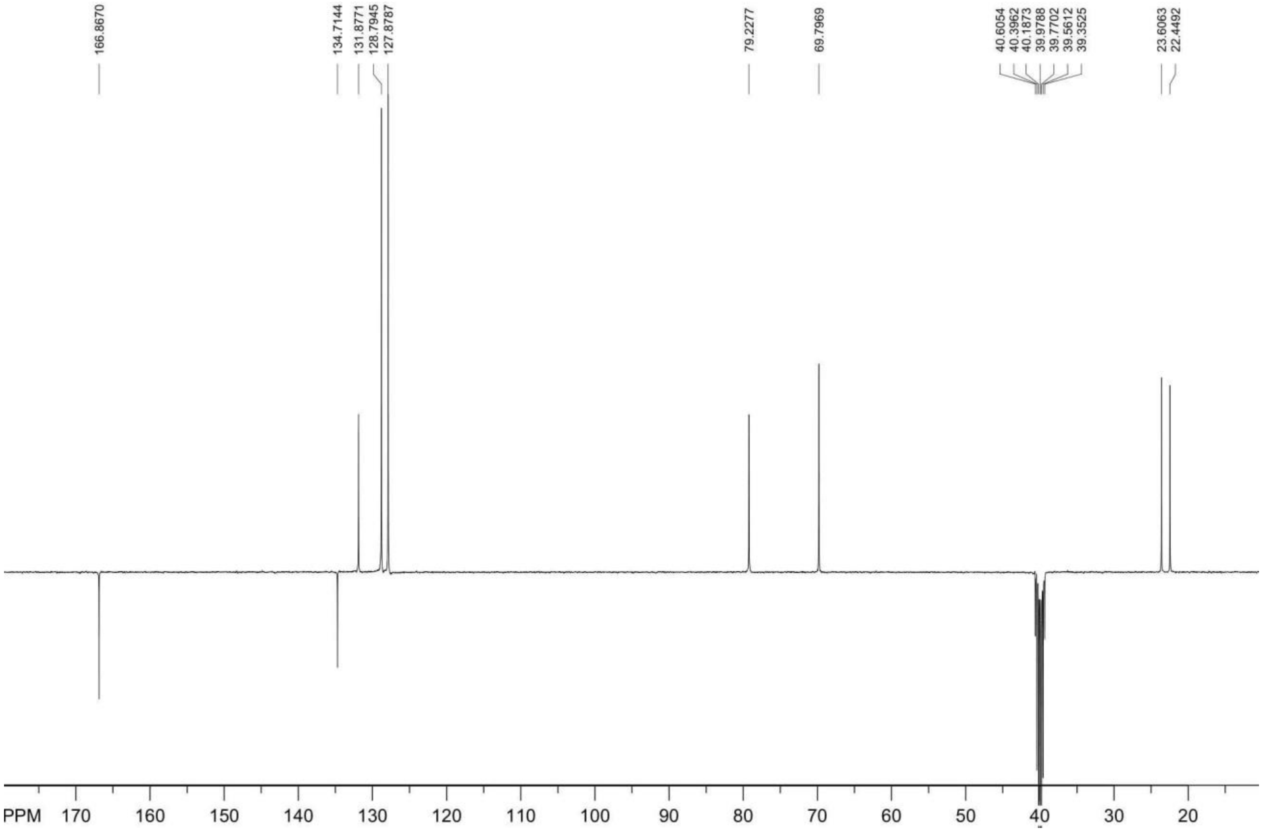

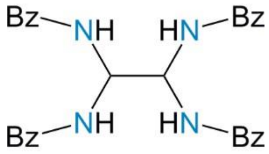

N,N',N",N"'-(Ethane-1,1,2,2-tetrayl)tetrabenzamide (**12**)

^{1}H (400 MHz) NMR (DMSO-d6, 24°C)

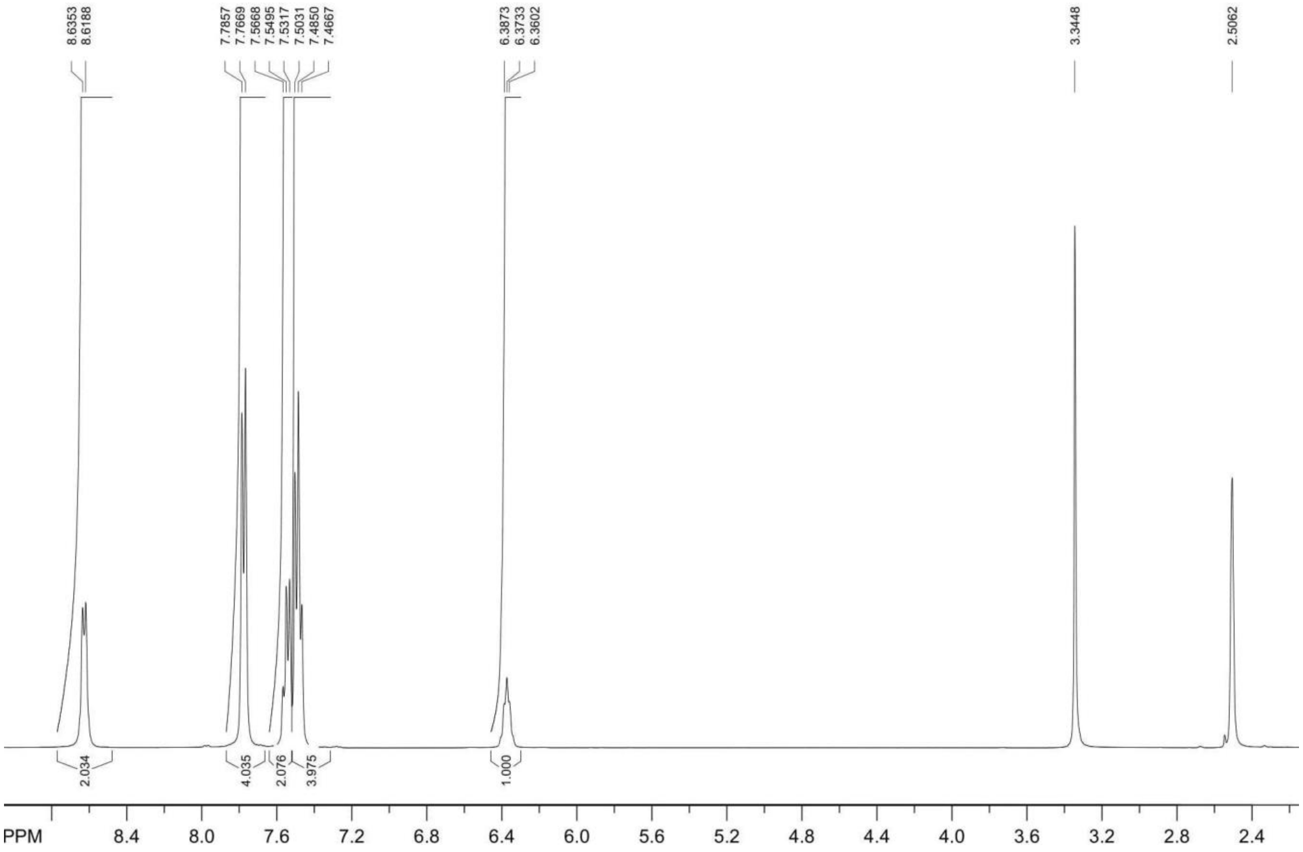

^{13}C (100 MHz) NMR (DMSO-d6, 24°C)

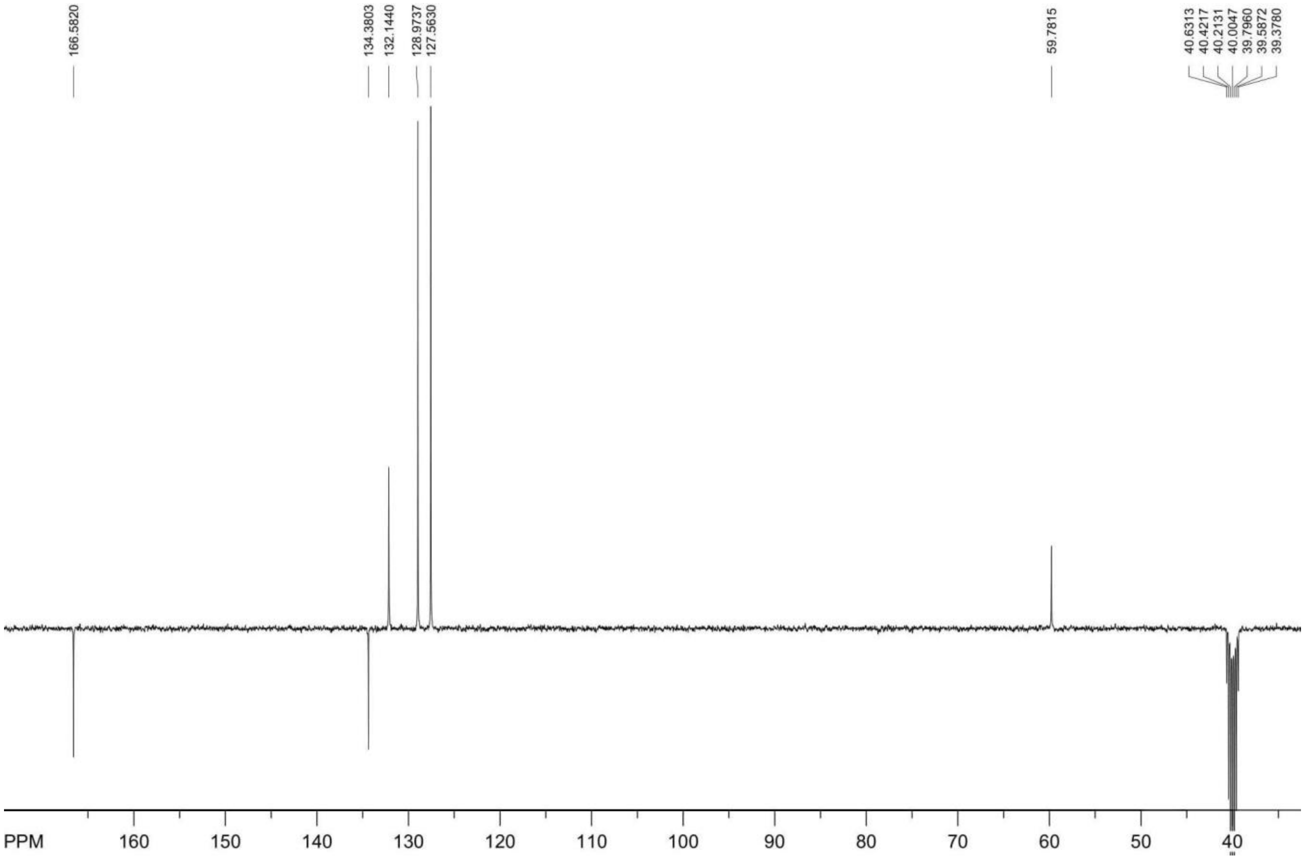

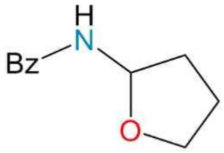

N-(Tetrahydrofuran-2-yl)benzamide (**13**)

^{1}H (400 MHz) NMR (DMSO-d6, 24ºC)

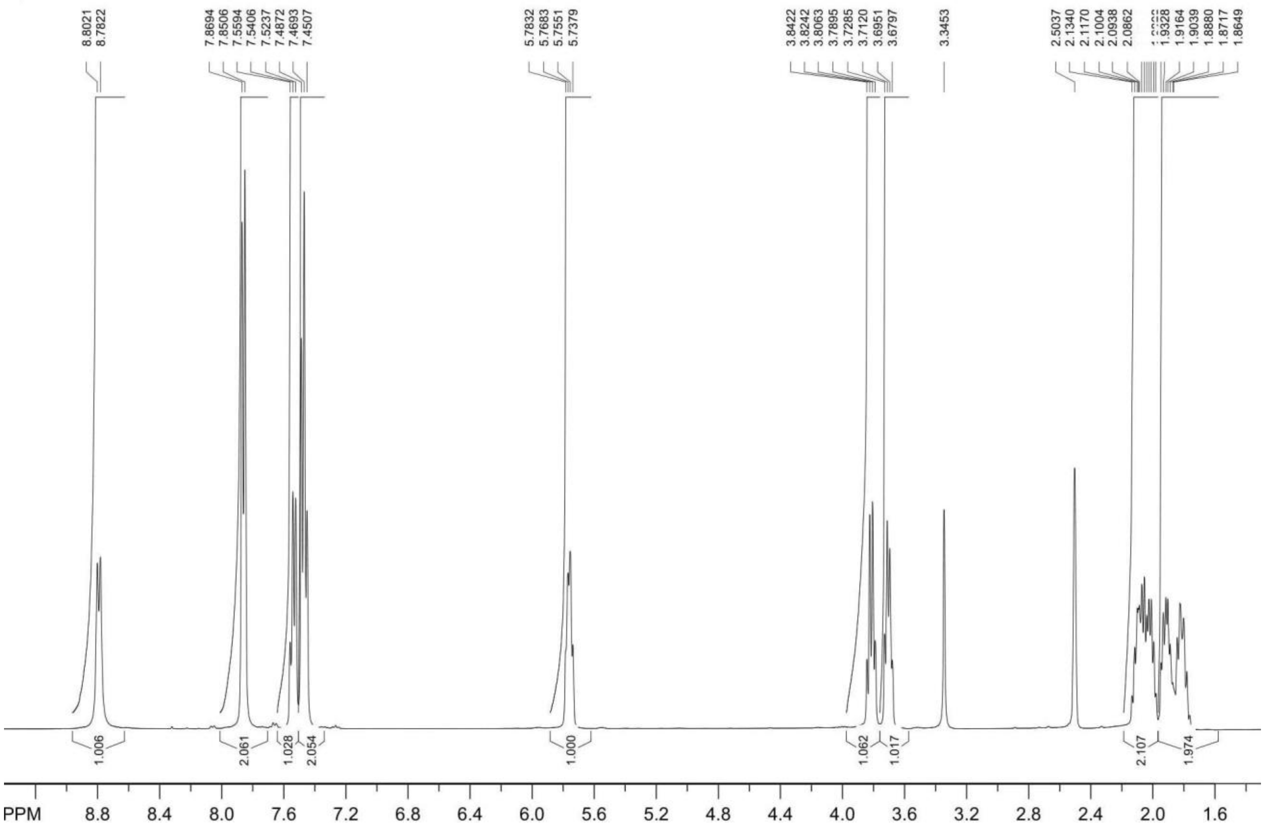

^{13}C (100 MHz) NMR (DMSO-d6, 24°C)

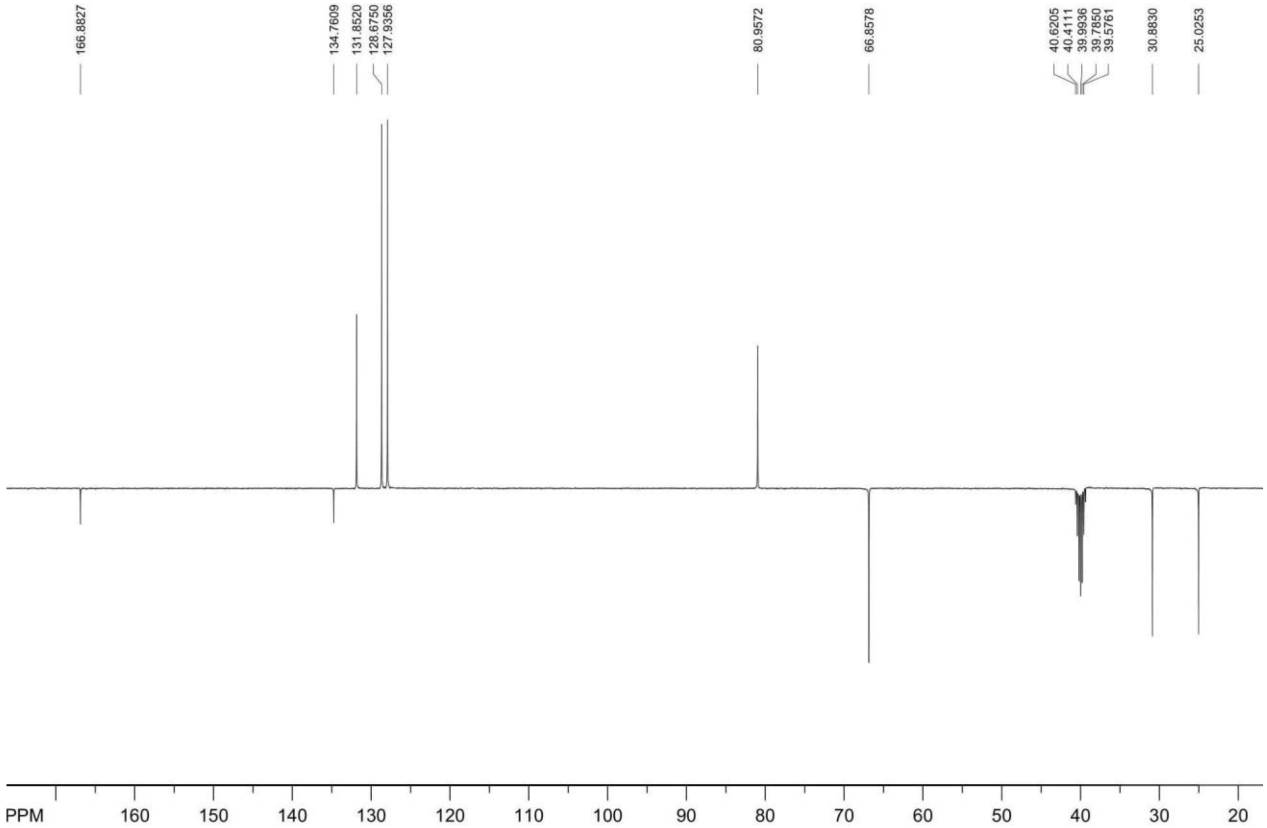

X-ray diffraction analysis data

Table 3 XRD data for compound **13**.

Empirical formula	$C_{11}H_{13}NO_2$
Formula weight	191.22
Crystal system	Orthorhombic
Space group	Pbcn
Unit cell dimensions *a* Å	22.735(2)
b Å	8.7951(7)
c Å	9.9496(6)
α º	90
β º	90
γ º	90
Volume $Å^3$	1989.5(2)
Z	8
Density (calcd.) $Mg.m^{-3}$	1.277
Abs. coefficient mm^{-1}	0.088
F(000)	816
Crystal size mm^3	0.02 x 0.08 x 0.90
Θ range for data collection °	2.5 - 27.5
Index ranges	$-29 \leq h \leq 29$, $-11 \leq k \leq 11$, $-12 \leq l \leq 12$
Reflections collected	25747
Independent reflections	2301 R(int)= 0.090
Completeness to θ %	99.9
Data / restraints / parameters	2301 / 0 / 130
Goodness-of-fit on F^2	1.00
Final R indices $I > 2\sigma(I)$	$R_1 = 0.0497$, $wR_2 = 0.1396$
Final R indices (all data)	$R_1 = 0.0947$, $wR_2 = 0.1674$
Largest diff. peak / hole $e.Å^{-3}$	0.27 / -0.23
CCDC	2108743

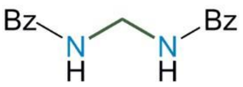

N,N'-Methanediyldibenzamide (**15**)

^{1}H (400 MHz) NMR (DMSO-d6, 24ºC)

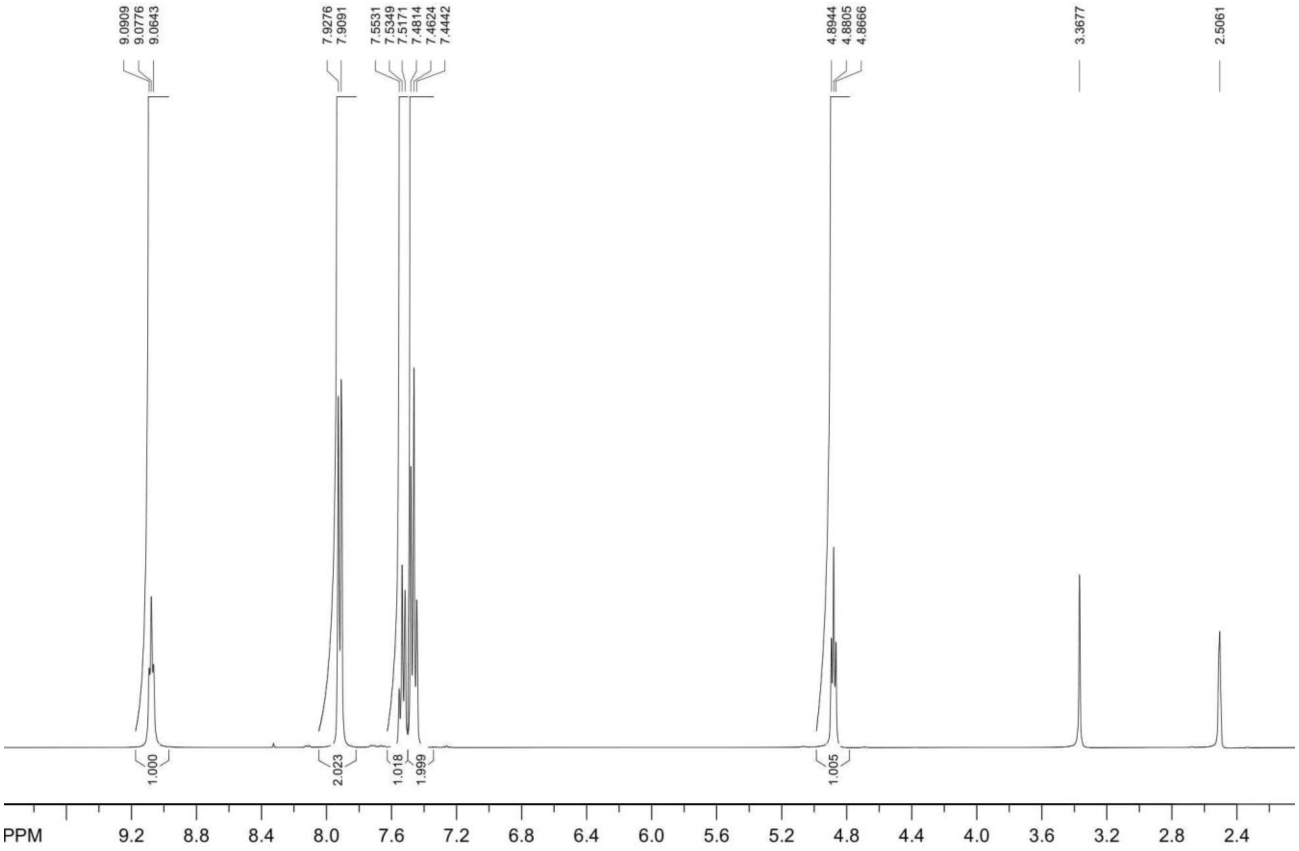

^{13}C (100 MHz) NMR (DMSO-d6, 24°C)

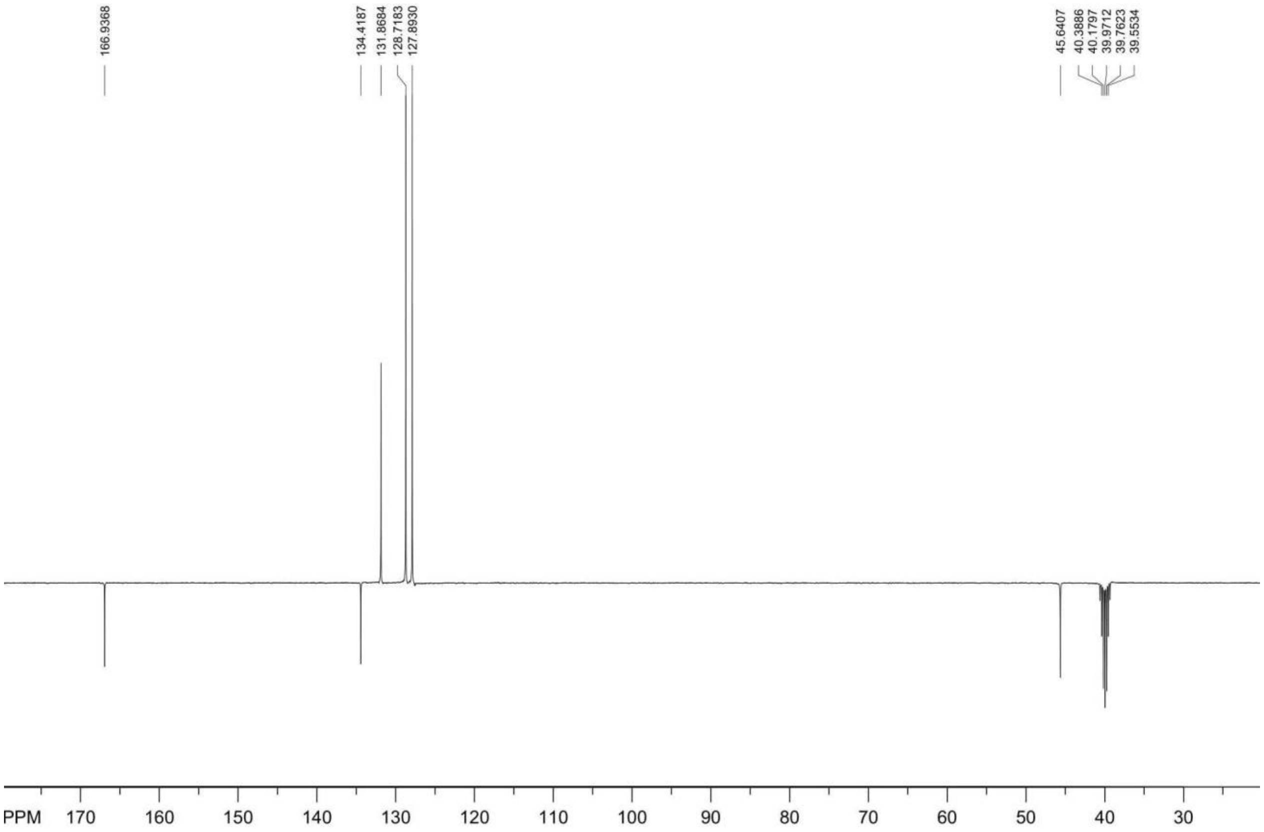

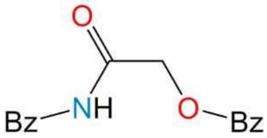

2-oxo-2-[(Phenylcarbonyl)amino]ethyl benzoate (**16**)

^{1}H (400 MHz) NMR (DMSO-d6, 24ºC)

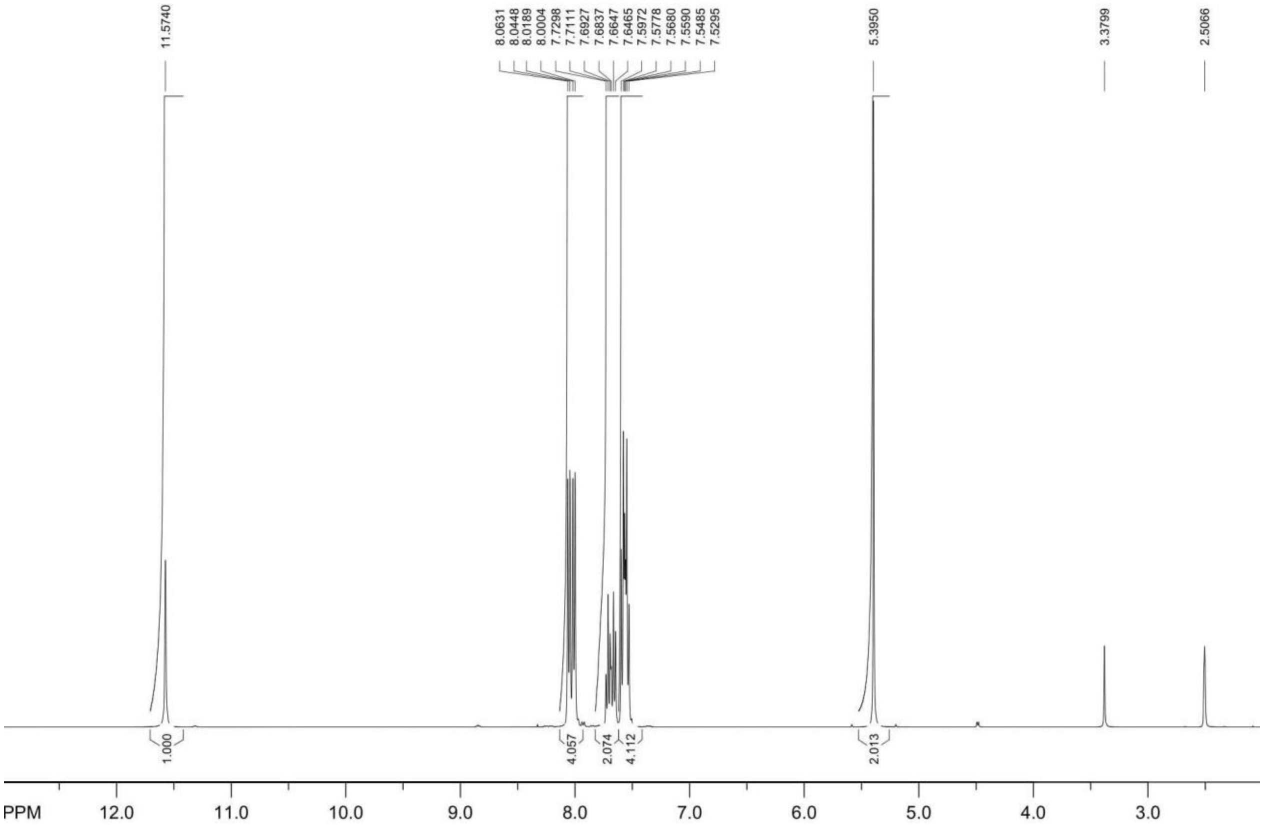

^{13}C (100 MHz) NMR (DMSO-d6, 24ºC)

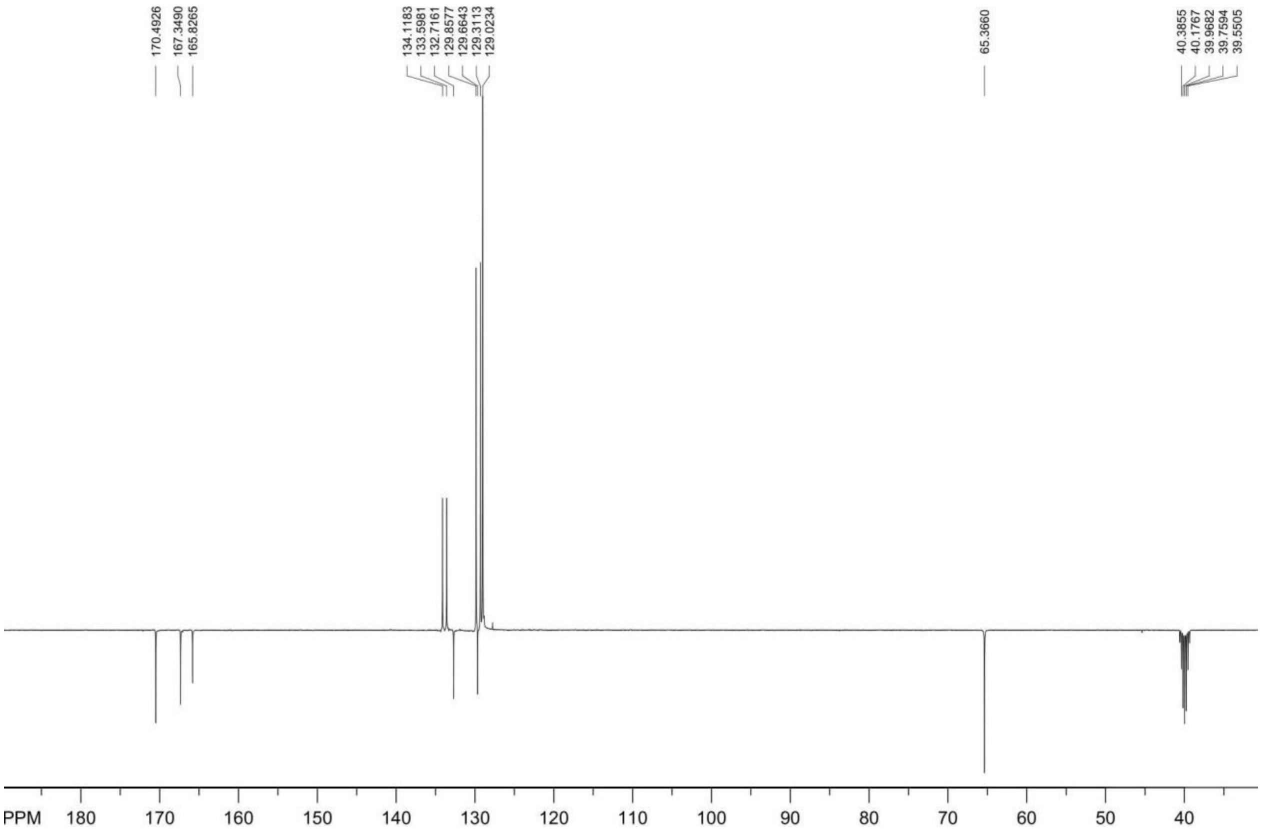

X-ray diffraction analysis data

Table 4 XRD data for compound **16**.

Empirical formula	$C_{16}H_{14}NO_4$
Formula weight	284.28
Crystal system	Monoclinic
Space group	C 2/c
Unit cell dimensions *a* Å	19.452(1)
b Å	5.1175(3)
c Å	27.952(2)
α °	90
β °	93.020(4)
γ °	90
Volume $Å^3$	2778.6(3)
Z	8
Density (calcd.) $Mg.m^{-3}$	1.359
Abs. coefficient mm^{-1}	0.099
F(000)	1192
Crystal size mm^3	0.04 x 0.20 x 0.70
Θ range for data collection °	2.1 – 26.1
Index ranges	$-24 \leq h \leq 24$, $-6 \leq k \leq 4$, $-34 \leq l \leq 34$
Reflections collected	14637
Independent reflections	2762 R(int) = 0.059
Completeness to θ %	99. 8
Data / restraints / parameters	2762 / 0 / 193
Goodness-of-fit on F^2	1.02
Final R indices $I > 2\sigma(I)$	R_1 = 0.0472, wR_2 = 0.1163
Final R indices (all data)	R_1 = 0.0635, wR_2 = 0.1273
Largest diff. peak / hole e.$Å^{-3}$	0.17, -0.22
CCDC	2108744

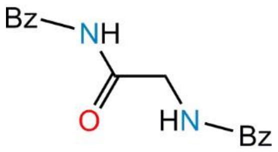

N,N'-(1-Oxoethane-1,2-diyl)dibenzamide (**17**)

^{1}H (400 MHz) NMR (DMSO-d6, 24ºC)

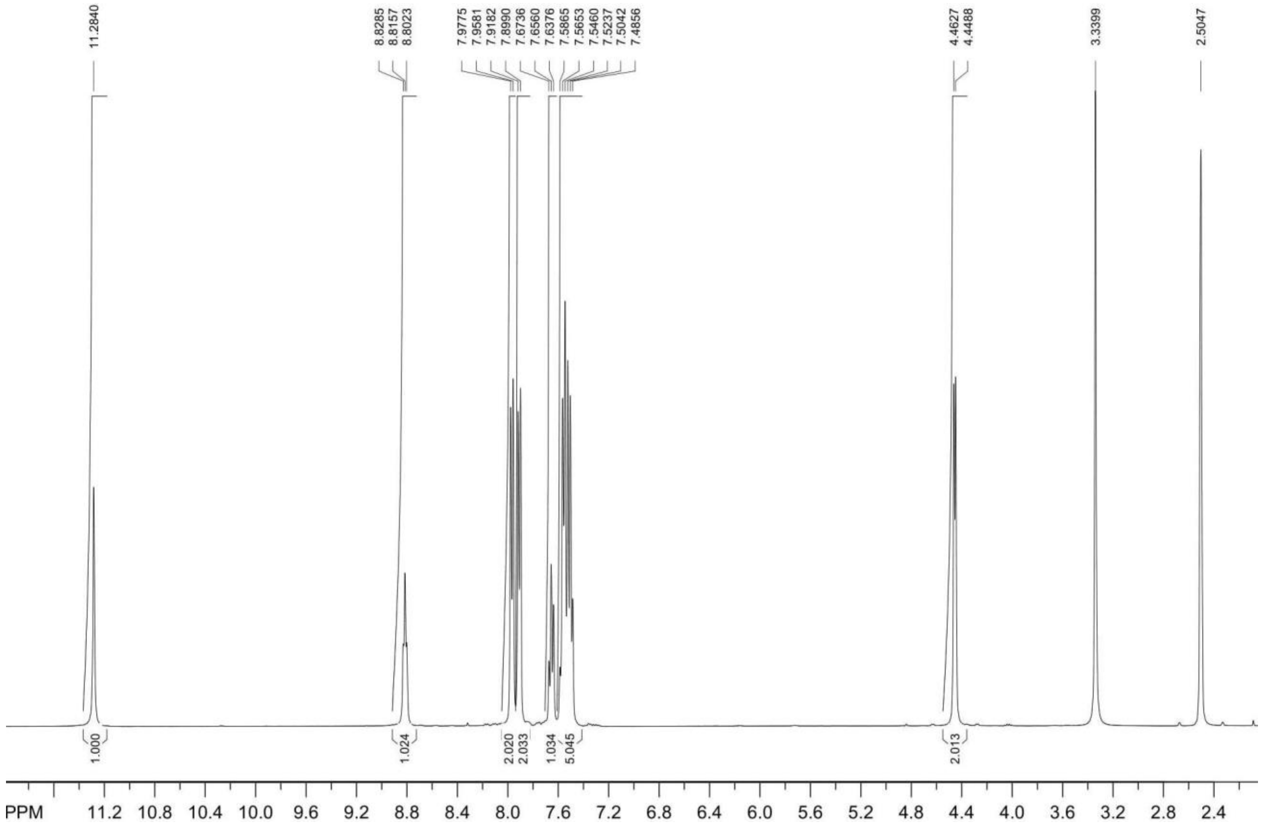

^{13}C (100 MHz) NMR (DMSO-d6, 24ºC)

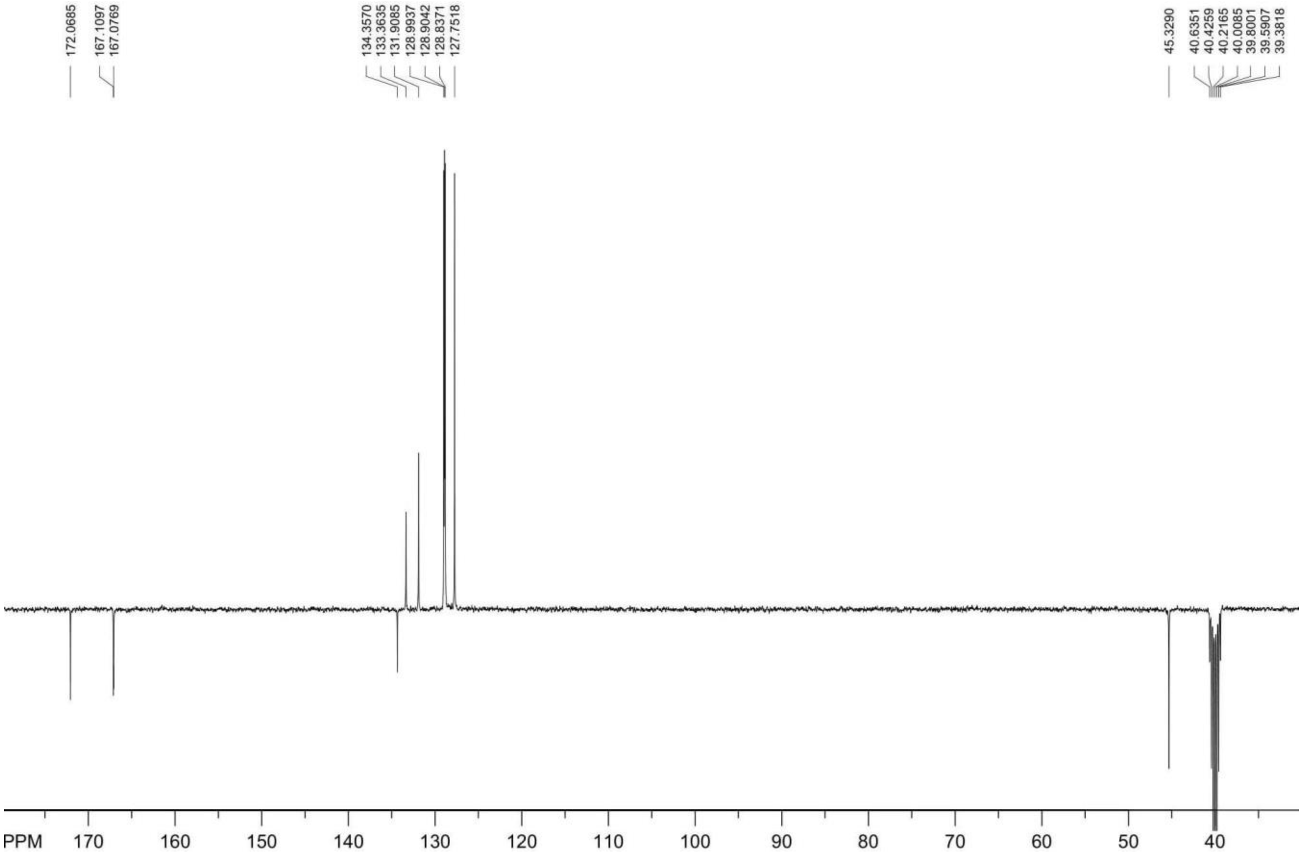

X-ray diffraction analysis data

Table 5 XRD data for compound **17**.

Empirical formula	$C_{16}H_{14}N_2O_3$
Formula weight	282.29
Crystal system	Monoclinic
Space group	$P2_1/c$
Unit cell dimensions *a* Å	16.4424(9)
b Å	4.9636(2)
c Å	17.466(1)
α °	90
β °	104.823(2)
γ °	90
Volume $Å^3$	1378.1(1)
Z	4
Density (calcd.) $Mg.m^{-3}$	1.361
Abs. coefficient mm^{-1}	0.096
F(000)	592
Crystal size mm^3	0.06 x 0.10 x 0.90
Θ range for data collection °	2.4 – 26.0
Index ranges	$-20 \leq h \leq 20, -6 \leq k \leq 6, -21 \leq l \leq 21$
Reflections collected	20012
Independent reflections	2720 R(int) = 0.039
Completeness to θ %	100.0
Data / restraints / parameters	2720 / 0 / 196
Goodness-of-fit on F^2	1.03
Final R indices $I > 2\sigma(I)$	R_1 = 0.0415, wR_2 = 0.0977
Final R indices (all data)	R_1 = 0.0710, wR_2 = 0.1174
Largest diff. peak / hole $e.Å^{-3}$	0.18, -0.19
CCDC	2108745

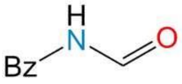

N-Formylbenzamide (**18**)

^{1}H (400 MHz) NMR (DMSO-d6, 24ºC)

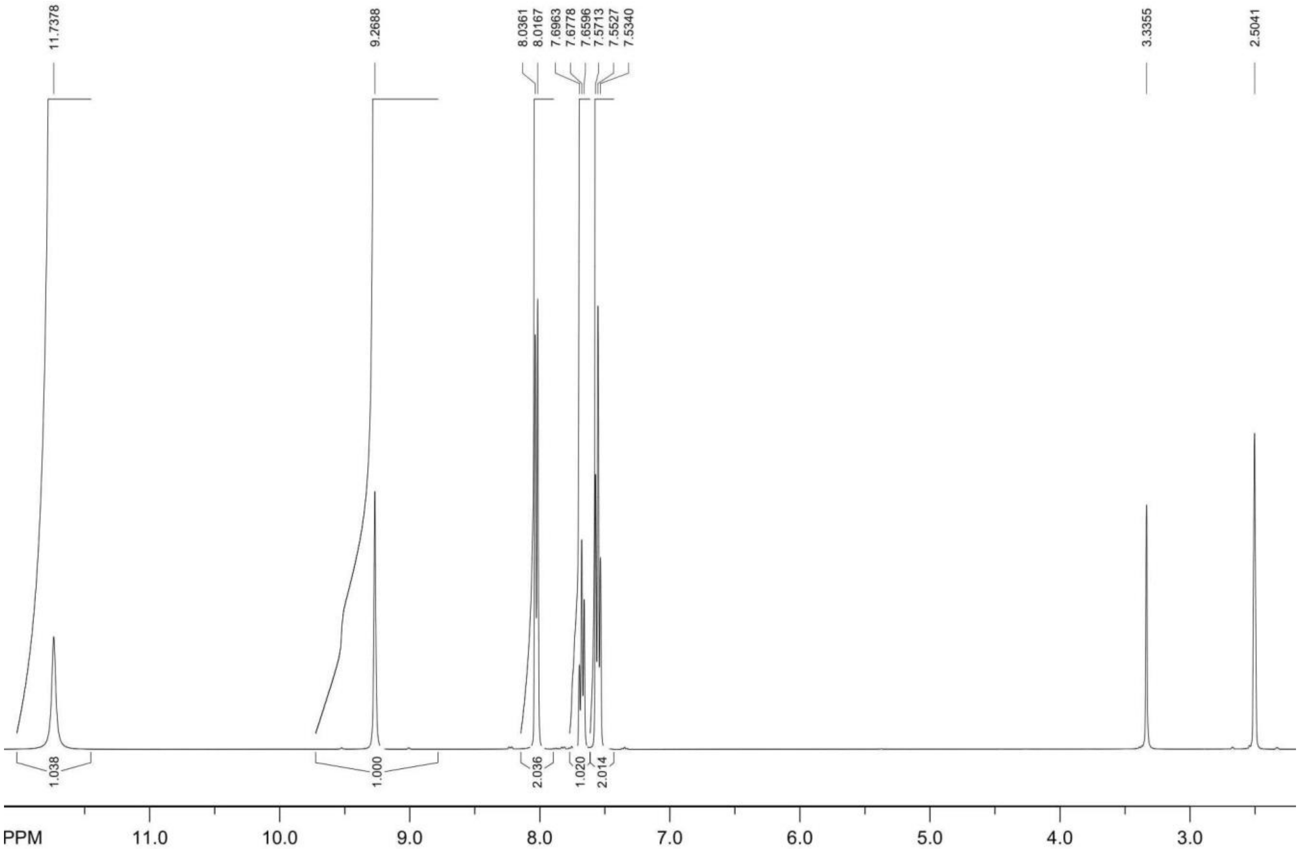

^{13}C (100 MHz) NMR (DMSO-d6, 24°C)

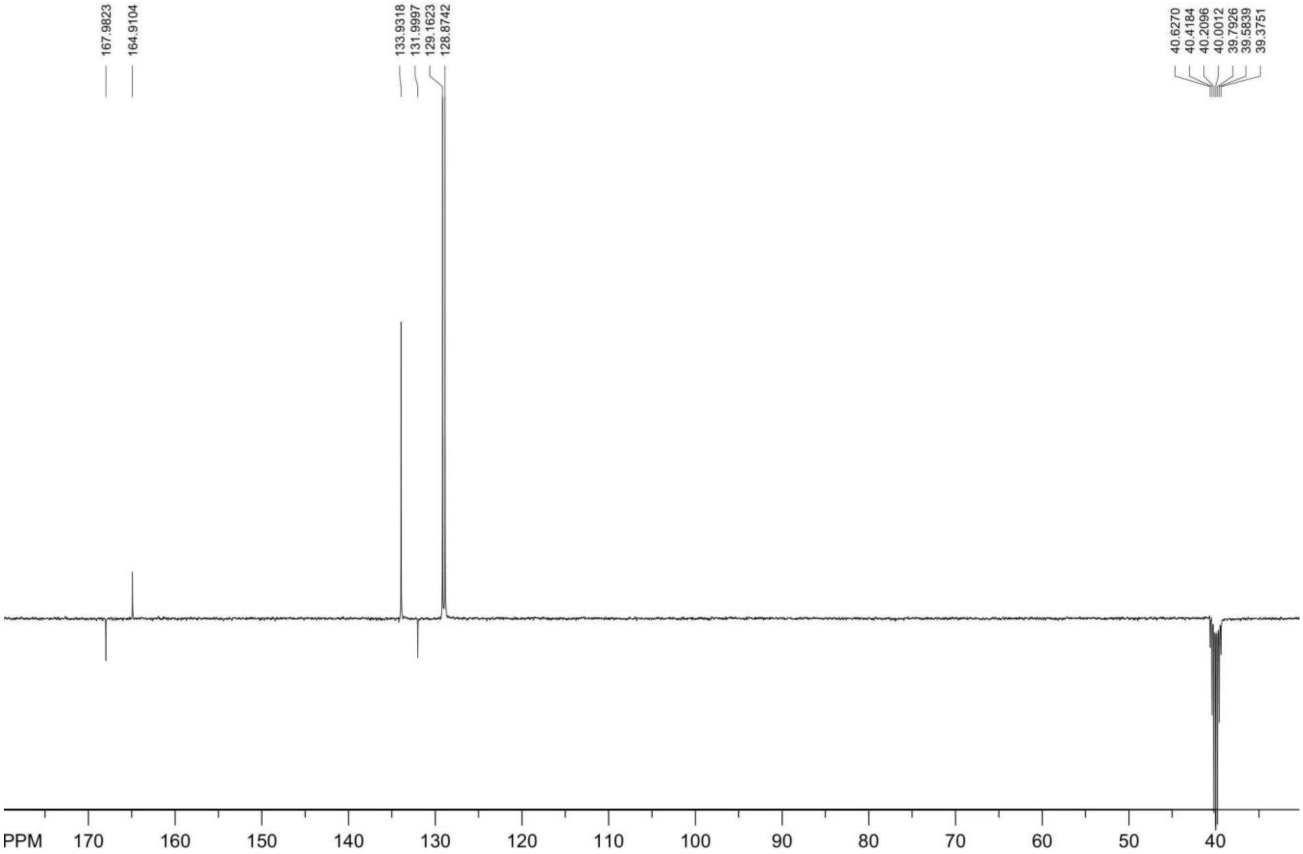

X-ray diffraction analysis data

Table 6 XRD data for compound **18**.

Empirical formula	$C_8H_7N_2O_2$
Formula weight	149.15
Crystal system	Monoclinic
Space group	$P2_1/c$
Unit cell dimensions *a* Å	11.643(1)
b Å	5.1501(4)
c Å	13.001(1)
α º	90
β º	110.118(4)
γ º	90
Volume Å^3	731.96(13)
Z	4
Density (calcd.) Mg.m^{-3}	1.353
Abs. coefficient mm^{-1}	0.099
F(000)	312
Crystal size mm^3	0.10 x 0.15 x 0.90
Θ range for data collection °	3.2 – 26.0
Index ranges	$-14 \leq h \leq 14$, $-6 \leq k \leq 6$, $-16 \leq l \leq 15$
Reflections collected	7436
Independent reflections	1448 R(int) = 0.053
Completeness to θ %	99.9
Data / restraints / parameters	1448 / 0 / 103
Goodness-of-fit on F^2	1.01
Final R indices $I > 2\sigma(I)$	$R_1 = 0.0407$, $wR_2 = 0.1097$
Final R indices (all data)	$R_1 = 0.0584$, $wR_2 = 0.1268$
Largest diff. peak / hole e.Å^{-3}	0.19/ -0.15
CCDC	2108746

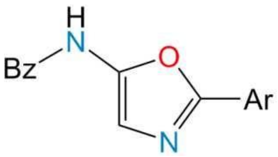

N-(2-Phenyl-1,3-oxazol-5-yl)benzamide (**19**)

^{1}H (400 MHz) NMR (DMSO-d6, 24ºC)

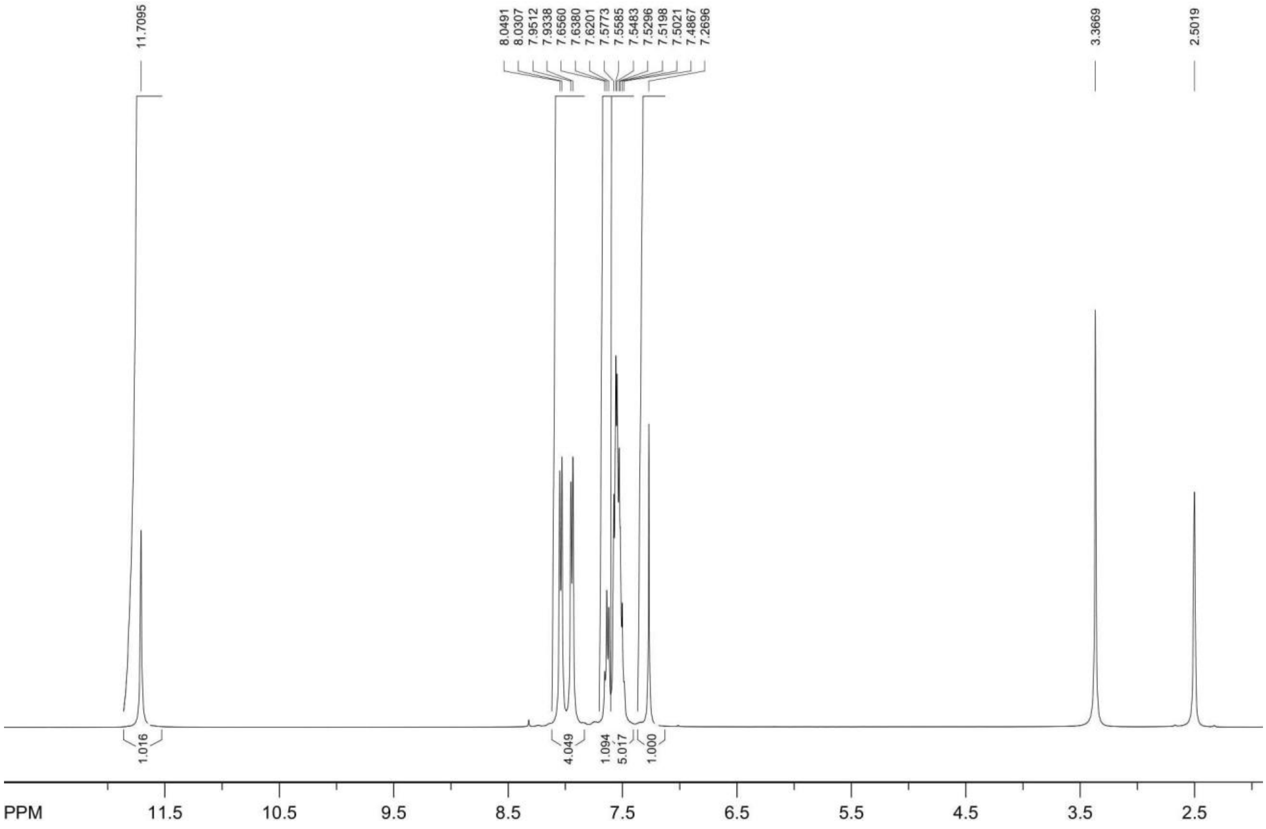

^{13}C (100 MHz) NMR (DMSO-d6, 24°C)

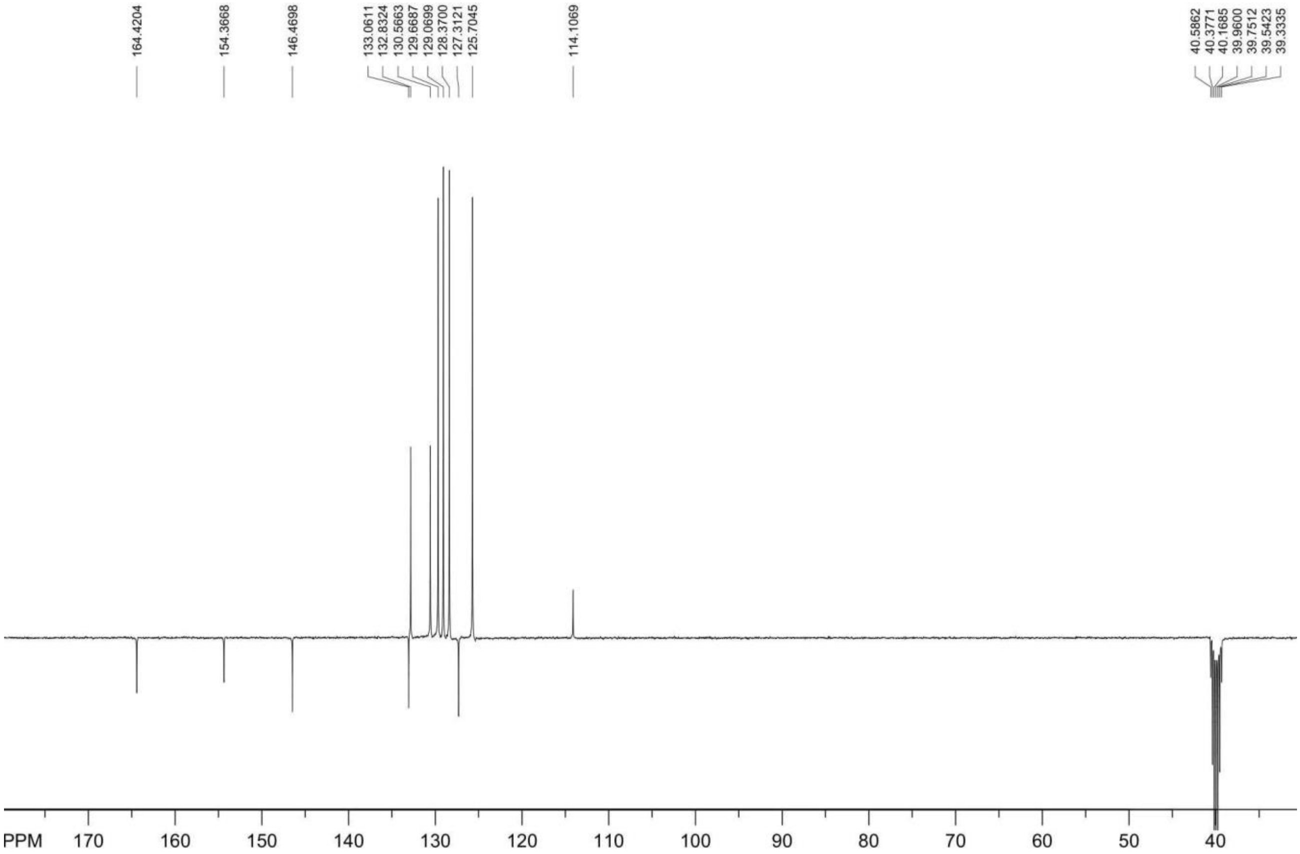

X-ray diffraction analysis data

Table 7 XRD data for compound **19**.

Empirical formula	$C_{16}H_{12}N_2O_2$
Formula weight	264.28
Crystal system	Monoclinic
Space group	$P2_1/c$
Unit cell dimensions *a* Å	11.3201(8)
b Å	12.4173(9)
c Å	10.3628(6)
α º	90
β º	113.811(2)
γ º	90
Volume $Å^3$	1332.7(2)
Z	4
Density (calcd.) $Mg.m^{-3}$	1.317
Abs. coefficient mm^{-1}	0.089
F(000)	552
Crystal size mm^3	0.06 x 0.10 x 0.80
Θ range for data collection °	2.6 – 27.5
Index ranges	$-14 \leq h \leq 14$, $-16 \leq k \leq 16$, $-13 \leq l \leq 13$
Reflections collected	26609
Independent reflections	3073 R(int) = 0.054
Completeness to θ %	100.0
Data / restraints / parameters	3073 / 0 / 184
Goodness-of-fit on F^2	1.02
Final R indices $I > 2\sigma(I)$	$R_1 = 0.0399$, $wR_2 = 0.0906$
Final R indices (all data)	$R_1 = 0.0693$, $wR_2 = 0.1062$
Largest diff. peak / hole $e.Å^{-3}$	0.13, -0.19
CCDC	2108747
